# Supplementary material for: Safety and efficacy of pulmonary physiotherapy in hospitalized patients with severe COVID-19 pneumonia (PPTCOVID study): A prospective, randomised, single-blind, controlled trial
Source: PLoS One. 2023 Jan 31;18(1):e0268428. doi: 10.1371/journal.pone.0268428 (PMC9888698; doi:10.1371/journal.pone.0268428)
Supplement: S1 Table — (DOCX) [file pone.0268428.s001.docx]

| **Type of Modification** | **Time of modification during the study** | **Reason(s)** |
| --- | --- | --- |
| Changing the "level of dyspnea" from primary to secondary outcome measurement | Before enrollment | Their role in the sample size calculation. The role of these outcomes were not important in this research to be considered as primary outcome measurement. |
| Changing the “rating perceived of exertion after the three-minute walk test" from primary to secondary outcome measurement |  |  |
| Changing the “Venous Blood PH" from primary to secondary outcome measurement |  |  |
| Consider the history of intubation due to COVID-19 pneumonia as exclusion criteria. | Before enrollment | When the research team was preparing to start enrolling the participants, they found that there are cases weaned from mechanical ventilator recently. Based on defined inclusion/exclusion criteria, they could include in the study because they could consider patients with severe COVID-19. As the main aim of this study was to evaluate the efficacy and safety of pulmonary physiotherapy in the severe stage of COVID-19 to determine that can this treatment improve patients with COVID-19 and prevent their deterioration?  Finally, the research team decided to consider the history of intubation due to COVID-19 as exclusion criteria. |

The permissions of all of the modifications were taken from the ethical committee of Tehran University of Medical Sciences.
