# Supplementary material for: Safety and efficacy of pulmonary physiotherapy in hospitalized patients with severe COVID-19 pneumonia (PPTCOVID study): A prospective, randomised, single-blind, controlled trial
Source: PLoS One. 2023 Jan 31;18(1):e0268428. doi: 10.1371/journal.pone.0268428 (PMC9888698; doi:10.1371/journal.pone.0268428)
Supplement: S2 Table — (DOCX) [file pone.0268428.s002.docx]

| **Cases** | | **Case 1** | **Case 2** | **Case 3** | **Case 4** | **Case 5** | **Case 6** | **Case 7** | **Case 8** | **Case 9** |
| --- | --- | --- | --- | --- | --- | --- | --- | --- | --- | --- |
| **Allocated group** | | Pulmonary physiotherapy group | Pulmonary physiotherapy group | Basic care group | Basic care group | Basic care group | Basic care group | Basic care group | Basic care group | Basic care group |
| **Age (years)** | | 45 | 59 | 74 | 43 | 63 | 69 | 72 | 70 | 69 |
| **Sex** | | Male | Female | Male | Male | Male | Female | Male | Male | Male |
| **Height (cm)** | | 172 | 160 | 183 | 171 | 172 | 161 | 170 | 172 | 169 |
| **BMI (kg/m^2^)** | | 25.7 | 31.3 | 24.2 | 26 | 24.2 | 25.3 | 22.5 | 22 | 28.4 |
| **Smoker** | | No | No | Yes | Yes | No | No | No | No | Yes |
| **Comorbidity** | | None | Cardiovascular disease | Diabetes | Renal failure | None | None | None | Cardiovascular disease | None |
| **Routine ventilation type** | | Partial Rebreather | Partial Rebreather | Partial Rebreather | CPAP | CPAP | Partial Rebreather | Partial Rebreather | Partial Rebreather | Partial Rebreather |
| **Signs or symptoms of excessive pulmonary secretion** | | Yes | No | No | No | Yes | No | No | No | No |
| **pO2 (mmHg)** | **Before** | 24 | 23.6 | 32.2 | 24.1 | 29.3 | 22.5 | 35 | 40.9 | 30.5 |
|  | **After** | - | 38.9 |  |  | 32.3 | 25.4 | 29 | 35.4 | 30.4 |
| **pCO2 (mmHg)** | **Before** | 44.6 | 44.4 | 44.4 | 50.1 | 43.3 | 26.4 | 43.6 | 48.1 | 37 |
|  | **After** | - | 38.5 | - | - | 43.1 | 43.6 | 38 | 52.9 | 43.1 |
| **Free air Spo2 (%)** | **Before** | 85 | 85 | 78 | 84 | 83 | 87 | 71 | 85 | 87 |
|  | **After** | - | 82 | - | - | 78 | 90 | 82 | 89 | 89 |
| **Distance of 3MWT (m)** | **Before** | 17.8 | 37.2 | 27 | 12.2 | 26 | 18 | 76 | 57.5 | 34 |
|  | **After** | - | 120.4 | - | - | 18.5 | 21 | 136 | 67.5 | 58 |
| **Level of Dyspnea** | **Before** | 6.8 | 8.4 | 5.4 | 7.8 | 5.2 | 5.4 | 5.2 | 6.3 | 2.5 |
|  | **After** | - | .7 | - | - | 4.3 | 3.7 | 3.6 | 5.7 | 2.2 |
| **RPE after Walking** | **Before** | 15 | 17 | 17 | 17 | 18 | 17 | 16 | 19 | 17 |
|  | **After** | - | 11 | - | - | 18 | 15 | 13 | 16 | 15 |
| **PH of peripheral venous blood** | **Before** | 7.22 | 7.48 | 7.35 | 7.36 | 7.39 | 7.51 | 7.39 | 7.48 | 7.48 |
|  | **After** | - | 7.58 | - | - | 7.33 | 7.6 | 7.5 | 7.54 | 7.36 |
| **Outcome** | | Intubated after second session with the pulmonary embolism diagnosis and expired after three days. | Re-hospitalized and finally died. | Intubated after day two due to ARDS and died seven days later. | Intubated after day one and expired after two days. | Intubated 11 days after the intervention and finally died three days later. | Intubated six days after the intervention and died seven days later | Intubated one day after intervention and expired ten days later. | Passed away with cardiac arrest diagnosis suddenly after three weeks. | Re-hospitalized |
