# Supplementary material for: Safety and efficacy of pulmonary physiotherapy in hospitalized patients with severe COVID-19 pneumonia (PPTCOVID study): A prospective, randomised, single-blind, controlled trial
Source: PLoS One. 2023 Jan 31;18(1):e0268428. doi: 10.1371/journal.pone.0268428 (PMC9888698; doi:10.1371/journal.pone.0268428)
Supplement: S1 Text — (DOCX) [file pone.0268428.s003.docx]

Tehran University of Medical Sciences

**Research Project/Thesis Proposal Form**

**Research Title:**

Efficacy of Chest Physiotherapy on Hospitalized Patients with Novel coronavirus 2019 Pneumonia

**Keywords:**

Pulmonary Physiotherapy– Novel Coronavirus 2019 – COVID-19 – Pneumonia

**Full Name of the Project Manager(s):**

Dr. Behrouz Attarbashi Moghadam

**School/Research Center:**

School of Rehabilitation

**Type of Research:**

Basic Applied/Clinical HSR

**This research is a:**

Student Thesis

TUMS Research Project

Joint Research Project

**Student’ Name (If it is a thesis):**

Mohammad Javaherian

**Project Description**

***If a thesis, specify the level:*** Undergraduate M.Sc. MPH PhD Postdoc.

**Type of Study: Please mark**

| Case series |  |
| --- | --- |
| Cross sectional |  |
| Case / control |  |
| Cohort |  |
| / interventional clinical trial |  |
| Experimental |  |
| Pharmaceutical Study |  |
| Implementation of a scientific/ executive Method |  |
| Test Review |  |
| Method Review |  |
| Qualitative |  |
| Health System Management Study |  |
| Software Design |  |

**Information about the Project Manager(s)**

- Full Name(s): Dr. Behrouz Attarbashi Moghadam
- Academic Rank: Associated Professor
- School/Research Center: School of Rehabilitation
- Department: Physiotherapy
- Research Location: Imam Khomeini Hospital Complex
- Expected Duration: Eight months
- Current Position and work location: Intensive care physiotherapist, Imam Khomeini Hospital Complex
- Work Phone Number: +989121883095
- Work Address: Pich-e-Shemiran, Enghelab Street, Tehran, Iran; Postal Code: 1148956111
- E-mail Address: Attarbashi@tums.ac.ir
- **Contact Number in case of emergency: +989129321391 (Mohammad Javaherian)**

**Research Project Team:** (Other supervisors, advisors, students, other partners)

|  | Full Name | Position and Academic Rank | Type of Involvement | E-mail address and Phone Number |
| --- | --- | --- | --- | --- |
| 1 | Azadeh Shadmehr | PhD., Professor | Advisor | Shadmehr@tums.ac.ir |
| 2 | Mohammad Taghi Beigmohammadi | MD., Associated Professor | Advisor | mbage46@gmail.com |

| 1. **Rationale and Backgrounds:**   The novel Coronavirus appeared in late December 2019 in Wuhan city in China and was regarded as the cause for coronavirus disease 2019 (COVID-19) in many patients (1). The virus that came to be known as severe acute respiratory syndrome coronavirus 2 (SARS-Cov-2) COVID-19, has already caused a pandemic now and has spread worldwide infecting more than 16 million people. Based on official report of Iran Ministry of Health, treatment, and Medical Education, emergence of this disease was confirmed on February 18th, 2020 in Iran. This virus is usually transmitted through airborne particles of the virus. Incubation period of the virus is reported to be 3-7 days and usually doesn’t exceed 14 days (2). Most common symptoms of the disease include fever, dry cough, breathlessness and fatigue. Some of the patients present with nasal congestion, runny nose and diarrhea. In more severe cases, the infection can cause pneumonia, acute respiratory distress syndrome (ARDS) and even death (3). In some studies, it has been reported that the complications of COVID-19 may even persist for a longer period of time (4).  Based on the current evidence, pathogenesis of COVID is about 3 percent (5). This virus has a high rate of transmission where every infected person may transmit the virus to 2.2 or 9.2 other people (6, 7). Thus, it is very important to implement proper interventional strategies to mitigate the spread of the novel Coronavirus.  Pulmonary physiotherapy is a simple, comprehensive, effective, harmless and safe method with the purpose of improving the respiratory symptoms in the patients with pulmonary diseases by educating effective cough, airway clearance techniques (ACTs), breathing exercise and omitting aggravating factors that can have an effective role in the management of respiratory conditions and cut down financial costs associated with the disease. Previous studies on the effectiveness of physiotherapy for patients with different respiratory conditions including asthma, chronic obstructive pulmonary disease show that these techniques may enhance level of physical activity, quality of life and aerobic capacity (8).  Due to the long duration of stay period in patients with COVID-19 at pre-intensive care and intensive care units (ICU), they may present physical, mental and pulmonary problems. Following discharge these patients have to also stay in quarantine which may even worsen the above conditions. Theoretically, it is assumed that the virus can cause the above mentioned issues for the patients for a long time and potentially restrict their physical activity (9). A cohort study with 109 survivors of severe acute respiratory syndrome (SARS) showed that although lung volume and spirometry values of the patients were totally normal after 6 months, carbon dioxide exchange and aerobic capacity was highly disrupted (10). It shows that viral pneumonia may cause fibrotic tissue formation in lungs.  So far, some researchers have studied the mechanism of pneumonia caused by the novel coronavirus 2019, but there is no single finding among them. One of the relatively powerful mechanisms currently used by pharmaceutical researchers was published by Franks et al. In connection with SARS-CoV in 2003 (11). According to their findings, the target cell of coronavirus in lung tissue is a pneumocyte type II, which is responsible for the production of surfactant and underlies the formation of type pneumocyte type I cell. On the other hand, according to the findings of Zhang et al., ACE2 receptors are the main SARS-CoV-2 receptor, which is present on the surface of pneumocyte type II cells (12). Therefore, reducing the secretion of surfactants and other proteoglycans can increase the stiffness of alveolar tissue, their tendency to collapse and ultimately reduce the level of perfusion of respiratory gases. Thus, pneumonia caused by COVID-19 as a viral pneumonia can cause biomechanical changes (especially viscoelastic properties) of alveolar tissue and interstitial space (9). These findings are completely consistent with Baig's view that the most important cause of COVID-19-induced pneumonia is decreased pulmonary perfusion and hypoxic vasoconstriction (13).  Because filtration of oxygen and carbon dioxide in the endothelial layer is disrupted in the case of COVID-19, it seems that oxygen therapy and other use of mechanical ventilation through different methods (Continuous Positive Airway Pressure (CPAP)), Bi-level Positive Airway Pressure (BiPAP), etc.), are suitable as non-pharmacological treatments for this disease.  Since there are several techniques in pulmonary physiotherapy that can lead to better ventilation at the alveolar surface, it appears that these methods can be considered as an effective treatment for the management of this disorder (14). The biomechanical hypothesis of these methods is the use of Creep phenomenon and other time-dependent properties of viscoelastic materials, during which the relative stiffness of alveolar tissue caused by insufficient secretion of surfactant can be reduced (15). On the other hand, according to the available reports, 34% of patients with pneumonia caused by COVID-19 present excessive pulmonary secretions (16) and it is possible that the application of ACTs can improve the patient's clinical symptoms.  Currently, the level of evidence regarding the effectiveness of respiratory physiotherapy in the inpatient phase is at a level that is more focused on previous limited findings in the face of Middle-East Respiratory Syndrome (MERS) or SARS and clinical experiences and the opinion of skilled professionals in this field. These levels of evidence are sufficient to suggest that pulmonary physiotherapy is theoretically an effective treatment, but it is not sufficient to be clinically proven to be effective for all patients.  On the other hand, for the past few months, every physiotherapist around the world have been facing the COVID-19 pandemic, two major questions have been on their minds regarding applying the physiotherapy to patients. First, can pulmonary physiotherapy be effective in acute conditions of the disease? and second, which kinds of pulmonary physiotherapy are indicated in different stages of acute COVID-19? A group of experts in this field argued that the role of physiotherapy in severe COVID-19 is only for patients with excess pulmonary secretions. The guideline published by the World Confederation of Physiotherapy are evidences of similar beliefs (17). When we look at the mechanism of pneumonia caused by COVID-19, and in particular the discussion of reducing surfactant secretion, we come to a biomechanical disorder that suggests that those breathing exercises that reduce alveolar tissue stiffness may be effective in improving symptoms and perhaps treating patients with COVID-19 even in severe stage. Our hypothesis does not rule out the possible effectiveness of ACTs in patients with excessive pulmonary secretions, but states that both patients with and without additional excessive pulmonary secretions may benefit from respiratory techniques to reduce alveolar tissue stiffness. According to this hypothesis, patients with coronavirus pneumonia should be divided into two groups; The first group is those who have excessive pulmonary secretions. These patients should first receive ACTs and then breathing exercises (such as diaphragmatic control, inspiratory hold technique or respiratory control) and the second group are patients whose clinical manifestations do not require ACTs, therefore for these patients only breathing exercises should be used. In a recently published randomized controlled trial (RCT), 72 elderly patients discharged from the hospital due to COVID-19 randomly allocated to physiotherapy and control groups. Researchers of this study showed that 12 sessions of physiotherapy (during 6 weeks) can significantly decrease anxiety, depression and level of disability and increase their quality of life and aerobic capacity (18). This study is the only published clinical trial in the field of pulmonary physiotherapy in the post-discharge phase of patients with COVID-19.  Therefore, we are currently facing gaps of knowledge in this field that can be filled with a series of research studies to be able to prescribe pulmonary physiotherapy to all patients with more confidence. The aim of this randomized controlled clinical trial study was to evaluate the safety and efficacy of pulmonary physiotherapy program in the hospitalization phase of patients with severe COVID-19 on respiratory findings, aerobic capacity, short-term and intermediate-term quality of life, mortality and re-hospitalization rates.   1. **The novelty of the subject:**   Given the widespread prevalence of SARS-CoV-2 worldwide and the development of widespread pneumonia in the community, it seems necessary to evaluate the short-term and intermediate-term therapeutic effects of pulmonary physiotherapy in the inpatient phase through a randomized controlled trial study. On the other hand, since there is currently a question among physiotherapists whether to use this treatment for all patients or only for patients with excessive pulmonary secretions, our second goal is to see the results of the treatment between the two groups of patients with and without excessive pulmonary secretion. The results of this research will be compared with other reports in this field and will be reviewed in detail in the form of a panel of experts in this field. According to the opinions of experts, an attempt is being made to develop a treatment protocol for respiratory physiotherapy in the hospitalization phase in patients with pneumonia caused by the COVID-19.   1. **Primary Literature Review:**   In 2013, AMBROSINO and colleagues reviewed physiotherapy techniques in the treatment of patients with ARDS. In this study, exercise therapy, mobilization, electrical stimulation, manual hyperinflation, percussion and in-ex sufflation methods were used. The results showed that in ARDS patients who are hospitalized in the intensive care unit, physiotherapy interventions should be started as soon as possible (14). In other words, the effectiveness of physiotherapy treatments depends on when they are initiated (19).  In 2017, Munshi et al., In a retrospective cohort study, examined the effects of physiotherapy interventions in the intensive care unit when using Extracorporeal Membrane Oxygenation (ECMO) for patients with acute respiratory syndrome ARDS. In this study, the daily activity level of these patients was coded using the ICU Mobility Scale. Sixty-one of the 107 ECMO-related patients had acute respiratory syndrome, and 82% underwent physical therapy in the intensive care unit. According to the results, physiotherapy during ECMO is completely safe and can be performed if performed by a specialist team (20).  In 2020, Yang et al. conducted a study of patients with COVID-19. In this study, the effects of pulmonary rehabilitation were investigated to prevent the spread of the virus, guide the patient to participate in a pulmonary rehabilitation program and to perform respiratory muscle exercises, excretion of secretions and to improve the mental health of patients. Breathing exercises, effective cough training, resistance training to strengthen the respiratory muscles, limb stretching exercises, strengthening exercises for the upper and lower limbs, bridging, cycling in the air were performed by the patient under the supervision of a therapist. Exercises were repeated twice a day and each exercise was repeated 15-20 times according to the patient's tolerance. Patients received oxygen when faced with hypoxia during the rehabilitation program. Patients with sputum cough performed all breathing exercises twice a day with 50 repetitions. The intensity of the exercises was adjusted according to the amount of resistance applied by the patients. According to the results of this study, physiotherapy treatments are safe for the patient, and since this treatment is not affected by time, place and facilities, the physiotherapist can perform the techniques at home or in the care unit. Also, due to high efficiency, it causes patient and staff satisfaction. One of the important points in the physiotherapy of these patients is the possibility of treatment via video and remote telephony, which is very important in preventing the spread of the disease (21). Unfortunately, this study was completely opinion-based and did not follow the design of a quantitative studies.  In 2020, Wang et al. in a review study have investigated previous evidences of viral pneumonias, SARS/MERS disease and different experts’ experiences within the rehabilitation field in order to review the current evidences to manage the physiotherapy of the COVID-19 patients. In this study, the goal of patients' respiratory rehabilitation was to improve the symptoms of shortness of breath, reduce stress levels, reduce problems caused by infection, reduce the level of disability and maintain functional capacity and quality of life of patients. In this study, patients' pulmonary rehabilitation techniques including respiratory discharge techniques, postural correction, breathing exercises and especially diaphragmatic breathing, stretching exercises, manual therapies and aerobic exercises were reviewed. According to these researchers, respiratory physiotherapy measures can start from acute conditions and continue for a period of time after discharge (22). Despite the excellent implementation of the primary article search method, the researchers in this article could not finally find a study related to the effects of respiratory physiotherapy in the inpatient phase on patients with SARS / MERS.  In 2020, Iannaccone et al. from Italy published a theory-based article on the situation at San Raffaele Hospital in Milan and their experiences with the rehabilitation of adults with Covid-19. According to the researchers, the length of hospitalization of Covid-19 patients in this hospital was 15 days. Since this long time can significantly reduce the patient's level of physical and mental ability, and the rehabilitation team of this hospital found that about 20% of patients are admitted to rehabilitation wards, rehabilitation professionals decided that patients after hospitalization in the acute wards enter the rehabilitation or quarantine departments. Patients were admitted to rehabilitation wards until they could regain their functional activities. The purpose of the rehabilitation program for Covid-19 patients in the acute and post-acute wards of this hospital is to increase the level of respiratory dynamics, to deal with musculoskeletal disorders, to reduce the incidence of hospitalization problems, to restore patients' cognitive and mental condition, to reduce disability and to improve quality of life at the time of discharge (23).  Physiotherapy interventions in the hospitalization phase of patients included 1) postural management, 2) interdisciplinary management of non-invasive ventilation, 3) improvement of patient’s active and inactive movements, and 4) determining the patient's respiratory and motor criteria for discharge. Patients admitted to the rehabilitation wards also received interventions of aerobic exercise, resistance, balance, improvement of daily activities and improvement of cognitive conditions (by a psychologist). The result of all these measures was to reduce the length of hospital stay to 10 days (23).  Also, all of these patients were monitored by the rehabilitation team through a remote rehabilitation system after being discharged from quarantine or rehabilitation wards.  In March 2020, Lazzeri et al. published a Position Paper on behalf of the Italian Association of Respiratory Physiotherapy, in which various aspects of respiratory physiotherapy in patients with Covid-19, including the possibility of transmitting the virus, various ventilation procedures, and patient posture changes, respiratory discharge techniques, ventilator function, weaning criteria, and possible problems in ICU patients were discussed (24).  Liu et al. From China in March 2020 published a randomized clinical trial study with a control group to evaluate the therapeutic effects of 6 weeks of respiratory rehabilitation on pulmonary function, quality of life (SF-36 questionnaire), Aerobic capacity (through a 6-minute walk test), physical activity (through functional independence measure), and mental function (by self-reported level of depression and anxiety) in elderly patients (over 65 years of age) discharged from hospital. Patients in the intervention group underwent 12 session of rehabilitation with 10 minutes duration for 6 weeks focusing on breathing exercises, effective coughing, aerobic and resistance training and stretching. The results of this study showed that this rehabilitation program can cause a statistically significant improvement compared to the control group in all the mentioned criteria (18).  In May 2020, Lu-Lu Yang and Ting Yang from China published a review study examining the types of possible rehabilitation interventions for hospitalized and discharged patients with Covid-19. According to the findings of this study, three evaluation tests, Breath-hold test, 1-minute step test and Squat, have been proposed as tests for patients with COVID-19. According to these researchers, the recommended techniques for these patients include diaphragmatic breathing (for 10 minutes at a frequency of 3 times a day), pulmonary discharge techniques (including exhalation exercises for 10 minutes and frequency once a day), Respiratory muscles strengthening, chest expansion exercises (including muscle stretching for 5 minutes at a frequency of 1 time per day), aerobic exercises (walking for 10-30 minutes with a target heart rate of 124 beats per minute, fatigue rate greater than 2 Borg scale and saturation of blood oxygen (more than 90%) and resistance training (using elastic band or weight) are the basis. Whether these exercises should be used for all patients with any condition or whether they should be optional is a question that has not been answered in this study (25).  In 2020, Thomas et al. published Clinical Practice Recommendation, reviewing various physiotherapy techniques for patients with Covid-19. This article addressed various aspects of physiotherapy for Covid-19 patients, including the number of physiotherapists required, physiotherapy equipment required in Covid-19 wards, care precautions for physiotherapy clinicians, and respiratory physiotherapy techniques. The interesting point of this study is that its authors, like the guideline published by the World Confederation of Physiotherapy, focus most of their techniques on pulmonary discharge measures, use of non-invasive positive pressure ventilation, patient start-up exercises, measures during mechanical ventilation in the intensive care unit, and active and non-active exercises and like other researchers, have not proposed aerobic exercise and pulmonary expansion exercise (17).  Robinson and Simpson from Canada published a Rapid literature review article examining the global Covid-19 pandemic situation and reviewing the latest goals of the global rehabilitation system for these patients. According to their findings, high levels of physical, cognitive and mental disorders in patients with COVID-19 can be expected and the rehabilitation system can play an important role in the process of care and return to normal life in patients with Covid-19. This rehabilitation program can also play an important role in helping maintain the administrative structure and economy of the world. They also mentioned the role of virtual rehabilitation program for this group of patients (26).  Based on a review of previous studies, we find that physiotherapy researchers have not yet conducted a study on the effectiveness of respiratory physiotherapy interventions in hospitalized patients due to pneumonia caused by COVID-19 in severe stage. So far, everything we have is based on the opinion of experts in this field, which is sometimes contradictory. In addition to providing a treatment method for patients in the inpatient phase based on clinical decision making, this study tries to evaluate its safety and effectiveness in the form of a randomized controlled trial.   1. **Definition of words and concepts:**   **Respiratory physiotherapy:**  Respiratory physiotherapy is a simple, comprehensive, effective, and safe treatment method that aims to improve patients' respiratory symptoms through effective cough education, airway clearance, respiratory exercises and elimination of aggravating factors, etc. It can be effective in the management of respiratory diseases and save on medical costs by improving the patient's condition (27).  In this study, different methods of respiratory physiotherapy in patients with pneumonia caused by the new coronavirus 2019 based on clinical decisions will be presented in two groups of breathing exercises and techniques for removing excessive pulmonary secretions.  **Novel Coronavirus 2019:**  SARS-CoV2 virus is a virus of the coronavirus family that causes COVID-19. The disease can manifest itself in the form of respiratory disorders (28). These disorders are generally pneumonia (lung infection) of the lungs  The patients of the present study are individuals with coronavirus pneumonia who are in the category of intensive care patients. The characteristics of these patients are mentioned in the form of inclusion and exclusion criteria.  **Mixed venous gas analysis:**  Mixed venous gas analysis is an alternative method for estimating blood gases (oxygen and carbon dioxide), acidity, blood oxygen saturation, and blood bicarbonate levels. In principle, this method is used for conditions where it is not possible to analyze arterial blood gases (29). According to the results of previous studies, the measurements obtained from venous gas analysis in patients with respiratory disorders have a high and significant correlation with the measurements obtained from arterial gas analysis (30).  In this study, venous gas analysis will be used for pulmonary function before and after the intervention period. VBG samples are taken at the hospital and will be sent immediately to the Arterial Blood Gasses Laboratory of Imam Khomeini Hospital. The transfer time of the sample from the ward to the laboratory will be less than 10 minutes. In order to control the sampling conditions, blood samples will be taken from the peripheral veins of the upper limb.  **Three-minute walk test:**  The 3-minute walk test is a simple, clinical test that provides a general and comprehensive answer to all systems involved in exercise, including the cardiovascular system, pulmonary system, musculoskeletal system, neuromuscular system, and metabolic system (31).  In this study, a 3-minute walk test will be taken from all patients before and after the interventions, taking into account their specific conditions. The criteria obtained from this test will include the amount of walking. Patients are also asked to report the amount of fatigue they feel at the end of the test on a based on Borg scale (between 6 and 20).  **Health-Related Quality of Life:**  According to the definition of the World Health Organization, health-related quality of life is considered as a general physical, mental and social condition of an individual that is not necessarily associated with or without disease (32).  In this study, the patient's quality of life is measured through the Short form-36 questionnaire, which is a health-related quality of life questionnaire.  **Blood oxygen saturation:**  Blood oxygen saturation percentage is a vital factor in the management and treatment of patient care. This percentage is the result of the number of oxygen-bound hemoglobin relative to total blood hemoglobin (33).  In this study, the percentage of blood oxygen saturation before and after interventions will be used. In order for us to have controlled conditions, this scale will be measured two minutes after the inhalation of open air and two minutes after the inhalation of oxygen by the Partial Rebreather or Reserve Mask with the patient in a certain position.   1. **General Aim:**   The aim of this RCT is to investigate the safety and efficacyof pulmonary physiotherapy on respiratory findings, aerobic capacity, quality of life, mortality and re-hospitalization rates in hospitalized patients with severe COVID-19. Also, by reviewing previous evidence and this RCT results and the opinion of the experts in this field, a respiratory physiotherapy guideline for managing hospitalized COVID-19 will be developed.   1. **Research Questions and Specific Objectives:**   **A: Research Questions:**  1. How much the mean of O2 and CO2 pressure, HCO3, PH, and oxygen saturation (SPO2) will be in control and intervention groups respectively in mixed venous blood sample before and after the physiotherapy intervention?  2. How much the mean of O2 and CO2 pressure, HCO3, PH, and SPO2 will be in severe COVID-19 patients with or without excessive pulmonary secretions in intervention group in mixed venous blood sample before and after physiotherapy?  3. How much the mean of SPO2 after two-minute breathing in free room air will be in control and intervention groups respectively before and after the physiotherapy intervention?  4. How much the mean of SPO2 after two-minute breathing in free room air will be in severe COVID-19 patients with or without excessive pulmonary secretions in intervention group before and after physiotherapy?  5. How much the mean of SPO2 after two-minute breathing trough partial rebreather will be in control and intervention groups respectively before and after the physiotherapy intervention?  6. How much the mean of SPO2 after two-minute breathing through partial rebreather will be in severe COVID-19 patients with or without excessive pulmonary secretions in intervention group before and after physiotherapy?  7. How much the mean of distance of three-minute walk test will be in control and intervention groups respectively before and after the physiotherapy intervention?  8. How much the mean of distance of three-minute walk test will be in severe COVID-19 patients with or without excessive pulmonary secretions in intervention group before and after physiotherapy?  9. How much the mean of rating of perceived exertion after three-minute walk will be in control and intervention groups respectively before and after the physiotherapy intervention?  10. How much the mean of rating of perceived exertion after three-minute walk will be in severe COVID-19 patients with or without excessive pulmonary secretions in intervention group before and after physiotherapy?  11. How much the mean of breathlessness will be in control and intervention groups respectively before and after the physiotherapy intervention?  12. How much the mean of breathlessness will be in severe COVID-19 patients with or without excessive pulmonary secretions in intervention group before and after physiotherapy?  13. How much the mean of short form-36 value (each domain, mental component summary score, and physical component summary score) will be in control and intervention groups respectively one and three months after intervention?  14. How much the mean of short form-36 value (each domain, mental component summary score, and physical component summary score) will be in severe COVID-19 patients with or without excessive pulmonary secretions in intervention group one month after intervention?  15. How much the related mortality rate will be in control and intervention groups respectively until one month after intervention?  16. How much the related mortality rate will be in severe COVID-19 patients with or without excessive pulmonary secretions in intervention group until one month after intervention?  **B: Specific Objectives:**  **B-1: Descriptive:**  1. Determining mean of O2, CO2, HCO3, PH, and SPO2 of mixed venous blood before and after applying interventions in experimental and control groups respectively in hospitalized patients with severe COVID-19.  2. Determining mean of O2, CO2, HCO3, PH, and SPO2 of mixed venous blood before and after applying pulmonary physiotherapy patients with/without excessive pulmonary secretion.  3. Determining mean of SPO2 after two-minute breathing in free room air before and after applying interventions in experimental and control groups respectively in hospitalized patients with severe COVID-19.  4. Determining mean of SPO2 after two-minute breathing in free room air before and after applying pulmonary physiotherapy patients with/without excessive pulmonary secretion.  5. Determining mean of SPO2 after two-minute breathing through partial rebreather before and after applying interventions in experimental and control groups respectively in hospitalized patients with severe COVID-19.  6. Determining mean of SPO2 after two-minute breathing through partial rebreather before and after applying pulmonary physiotherapy patients with/without excessive pulmonary secretion.  7. Determining mean of distance of three-minute walk test before and after applying interventions in experimental and control groups respectively in hospitalized patients with severe COVID-19.  8. Determining mean of distance of three-minute walk test before and after applying pulmonary physiotherapy patients with/without excessive pulmonary secretion.  9. Determining mean of rating of perceived exertion after three-minute walk test before and after applying interventions in experimental and control groups respectively in hospitalized patients with severe COVID-19.  10. Determining mean of rating of perceived exertion after three-minute walk test before and after applying pulmonary physiotherapy patients with/without excessive pulmonary secretion.  11. Determining mean of breathlessness before and after applying interventions in experimental and control groups respectively in hospitalized patients with severe COVID-19.  12. Determining mean of breathlessness before and after applying pulmonary physiotherapy patients with/without excessive pulmonary secretion.  13. Determining mean of short form-36 value (each domain, mental component summary score, and physical component summary score) one and three months after applying interventions in experimental and control groups respectively in hospitalized patients with severe COVID-19.  14. Determining mean of short form-36 value (each domain, mental component summary score, and physical component summary score) one and three months after applying pulmonary physiotherapy patients with/without excessive pulmonary secretion.  15. Determining the related-mortality rate until one and three months after applying interventions in experimental and control groups respectively in hospitalized patients with severe COVID-19.  16. Determining the related-mortality rate until one and three months after applying pulmonary physiotherapy patients with/without excessive pulmonary secretion.  **B-2: Analytic:**  1. Comparing the mean of O2 and CO2 pressure, HCO3, PH, and SPO2 of mixed venous blood before and after applying intervention in each experimental or control groups in hospitalized patients with severe COVID-19 with and without considering baseline variable as a covariate.  2. Comparing the mean of O2 and CO2 pressure, HCO3, PH and SPO2 of mixed venous blood before and after applying pulmonary physiotherapy in hospitalized patients with COVID-19 with/without excessive pulmonary secretion.  3. Comparing the mean of SPO2 after two-minute breathing in free room air before and after applying intervention in each experimental or control groups in hospitalized patients with severe COVID-19 with and without considering baseline variable as a covariate.  4. Comparing the mean of SPO2 after two-minute breathing in free room air before and after applying pulmonary physiotherapy in hospitalized patients with COVID-19 with/without excessive pulmonary secretion.  5. Comparing the mean of SPO2 after two-minute breathing through partial rebreather before and after applying intervention in each experimental or control groups in hospitalized patients with severe COVID-19 with and without considering baseline variable as a covariate.  6. Comparing the mean of SPO2 after two-minute breathing through partial rebreather before and after applying pulmonary physiotherapy in hospitalized patients with COVID-19 with/without excessive pulmonary secretion.  7. Comparing the mean of distance of three-minute walk test before and after applying intervention in each experimental or control groups in hospitalized patients with severe COVID-19 with and without considering baseline variable as a covariate.  8. Comparing the mean of distance of three-minute walk test before and after applying pulmonary physiotherapy in hospitalized patients with COVID-19 with/without excessive pulmonary secretion.  9. Comparing the mean of rating of perceived exertion after three-minute walk test before and after applying intervention in each experimental or control groups in hospitalized patients with severe COVID-19 with and without considering baseline variable as a covariate.  10. Comparing the mean of rating of perceived exertion after three-minute walk test before and after applying pulmonary physiotherapy in hospitalized patients with COVID-19 with/without excessive pulmonary secretion.  11. Comparing the mean of breathlessness before and after applying intervention in each experimental or control groups in hospitalized patients with severe COVID-19 with and without considering baseline variable as a covariate.  12. Comparing the mean of breathlessness before and after applying pulmonary physiotherapy in hospitalized patients with COVID-19 with/without excessive pulmonary secretion.  13. Comparing the mean of short form-36 value (each domain, mental component summary score, and physical component summary score) before and after applying intervention in each experimental or control groups in hospitalized patients with severe COVID-19 with and without considering baseline variable as a covariate.  14. Comparing the mean of short form-36 value (each domain, mental component summary score, and physical component summary score) before and after applying pulmonary physiotherapy in hospitalized patients with COVID-19 with/without excessive pulmonary secretion.  15. Comparing the frequency of related-mortality before and after applying intervention in each experimental or control groups in hospitalized patients with severe COVID-19 with and without considering baseline variable as a covariate.  16. Comparing the frequency of related-mortality before and after applying pulmonary physiotherapy in hospitalized patients with COVID-19 with/without excessive pulmonary secretion. |
| --- |

| 1. **Research Design and Methods:**   **I. Study design:**  Single-blinded Randomized Controlled Trial and conducting a therapeutic protocol through Delphi method  **II. Subjects:**   - **Study Population:**   Hospitalized patients with severe COVID-19 pneumonia at Imam Khomeini Hospital Complex   - **Inclusion/exclusion criteria**   **Inclusion Criteria:**   1. Patients with COVID-19 pneumonia confirmed by RT-PCR test and diagnostic radiology. 2. 18 to 75 years old 3. Non-intubated patients 4. Patients with full consciousness 5. Be able to walk and perform exercises. 6. O2 Saturation < 88% when free air breathing. 7. be able to write and read in Farsi   **Exclusion Criteria:**   1. Any type of musculoskeletal disorder disabling patient to participate in the study. 2. Intubation during the period of intervention 3. Patients' dissatisfaction to continue the study for any reason. 4. Unable to complete at least three sessions of physiotherapy.   **III. Sample Size:**  Target variable: SPO_2_  Clinical significancy of SPO_2_: 4%  Standard Deviation: 8.8%  Type I error: .01  Power: 80%  Sample size: 13 per group  Final sample size after considering possible attrition: 20 per group  **IV. Methods:**  After receiving permission from ethical committee of Tehran University of Medical Sciences, patients admitted to pre-intensive care units and intensive care units with definite COVID-19 diagnosis who have severe clinical symptoms and aren’t intubated will be invited to participate in this study. After receiving informed consent, evaluation will begin. In this procedure, all outcome measurements including mixed venous blood gases, SpO_2_ in free air room and breathing through partial rebreather, distance of 3-minute-walk test and its perceived exertion and patients’ breathlessness will be measured. Before applying intervention, the investigator will conduct randomization through closed envelopes given to him/her. In this study, randomization will be done using Block balanced randomization method. This randomization method is the best way to homogenize study groups (34). The investigator who is responsible for applying intervention will allocate participants to the groups based on allocation concealment principles (35).  The duration of this study will be 3 days. This time was selected based on the mean of patients’ duration of stay in pre-intensive care units. This is an add-on randomized control trial study. Patients in both groups will receive consultation by physiotherapist with emphasis on breathing control and general exercises during hospitalization benefits of coughing and instruction on using incentive spirometer.  **Experimental group intervention:**  In this phase, patients receive respiratory physiotherapy two times a day (totally 6 sessions). This procedure includes patient evaluation, clinical decision making and applying interventions (27).   1. Evaluation:  - Vital signs (HR, RR, BP) - ECG - Drug chart - Body temperature - VBG or ABG - Pulmonary sounds (?) - Pulmonary palpation - SpO_2_ - Clinical imaging (CT-Scan or X-ray)  1. Clinical decision making:   Considering therapist’s clinical examination and findings in each session, 2 treatment protocols will be considered for the patient:  Patients with Excessive Pulmonary Secretions  At first, based on therapist’s diagnosis and clinical decision making, ACTs will be administered for the patient. This treatment continues as long as airway secretions signs will be improved significantly. After applying these techniques, the patient will be treated with indicated dose of breathing control and diaphragmatic breathing in patients without airway secretion.  These techniques will include active cycle of breathing technique (ACBT), Autogenic Drainage, percussion, vibration, postural drainage and effective cough technique (27). All of these techniques will be performed when patients receive oxygen. These patients will then receive inspiratory hold technique with emphasis of diaphragmatic breathing.  Patients without airway secretions  These patients will receive inspiratory hold technique with emphasis of diaphragmatic breathing.  These techniques will be done in 3 sets with 10 repetitions:  **First set:** hold breath for 3 seconds, rest for 6 seconds  **Second set:** hold breath for 6 seconds, rest for 12 seconds  **Third set:** hold breath for 10 seconds, rest for 20 seconds  From the third session onwards, the patient will be asked to walk in the ward area (with/without using oxygen) after receiving treatment, walk for a maximum of 6 minutes. Factors which determine the end of the walk are:   - 1. O_2_ saturation reaches 80%   2. Rating of perceived exertion based on Borg scale: 11-13 (36)   In the end, instructions will be given to patient about effective cough (in case of any secretion), upper respiratory muscle relaxation and diaphragmatic breathing.  **Data collection method and tools:**  **Primary Outcome Measurements:**   - VBG analysis: Blood sample will be taken from a peripheral vein after 2-minute breathing in free room air. Factors such as PH, PvCO_2_, PvO_2_ and O_2_ saturation will be extracted from this analysis. As non-intubated patients with COVI-19 mostly don’t have arterial line, sampling from artery and performing arterial blood gas will be every painful and unethical. Apart from that, these patients have a respiratory (not cardiac) condition which based on previous studies there is a significant correlation between arterial and venous analysis (30). - 3-minute-walk test: Based on previous studies, this test is a simple cheap way with submaximal intensity that can be used in either in and out-patient setting. Also, there is a significant correlation between this and 6-minute-walk test (19, 20).   The patient will be asked to walk a smooth pathway with maximum speed possible for him/her in 3 minutes. Patient must have comfortable footwear during participating in this test. The patient will be instructed to stop walking and inform by the to stop walking if any sign of pain, inability, dyspnea and etc. occurred. During the test, the investigator won’t encourage the patient to increase his/her speed. After finishing the test, the patient is asked to stop and the investigator will measure the travelled distance.  If any cardio-vascular consequences or conscious disturbance occurs during operating this test, the physiotherapist will call for the physicians.   - Measuring O_2_ saturation after 2-minute breathing via partial rebreather: in this test, the patient will be asked to be in Fowler position for 2 minutes. A partial rebreather mask will be fixed on his/her face. Then the patient is asked to breathe deeply via the mask. After 2 minutes, the O_2_ saturation will be measured with a pulse oximeter.   **Secondary Outcome Measurements:**   - Level of breathlessness: At first, a visual analogue scale (VAS) image is given to the patient. The patient will be instructed that zero stands for no breath shortness and 10 shows maximum breath shortness. The patient will be asked to mark his/her feeling of breath shortness during past 24 hours on the paper with a pen. - Rating of perceived exertion based on Borg scale: On this scale 6 is very mild trouble and 19 is severe trouble during or after doing activities. The patient will be instructed to state his/ her dyspnea using this scale. - Health-related quality of life: One month after finishing the interventions, the patient will be contacted and will be asked to fill the short form-36 quality of life questionnaire. - Mortality rate: Based on the patient’s medical record, the patients’ related-mortality rate until one month will be evaluated   **Treatment protocol development:**  One of the main aims of this study is to develop a treatment protocol for managing (evaluation and treatment) hospitalized patients because of COVID-19 pneumonia on behalf of Tehran university of medical sciences. To achieve this goal, after finishing this RCT, a systematic review of all the published studies related to the physiotherapy program of these patients in the inpatient phase will be provided. Search and data extraction in this systematic review will be according to PRISMA standard. After data extraction, assessing the studies quality and this RCT report, the result will be presented in an expert panel including physiotherapists and other related medical fields experts to express their views. The main investigator will gather and classify all the views. Considering the level of their evidence and quality, a draft for the protocol text will be written. This text will be presented again in the expert panel for evaluating its content validity. Another version of the draft will be provided after applying the changes made by the expert panel and will be given to 5 physiotherapists visiting patients with COVID-19. They will be asked to express their views on the comprehension and unclear paragraphs (face validity). the output of these steps will provide physiotherapy protocol for hospitalized patients with COVID-19 induced pneumonia. Also, in order to ask the expert panel about their agreement to different parts of recommended protocol based on Delphi method. |
| --- |

1. **Estimated total time to complete the research (in months):**

Eight months

**8- Research Timeline Table:**

Prepare a list of the activities planned for the research proposed. Mark with X the appropriate cells to reflect the time (each cell represents one month) and duration of each activity.

An example of activities is provided in the first three rows.

|  | **Activities** | **Duration of the activity** | | | | | | | | | | | | | | | | | | | | | | | | | | | | | |
| --- | --- | --- | --- | --- | --- | --- | --- | --- | --- | --- | --- | --- | --- | --- | --- | --- | --- | --- | --- | --- | --- | --- | --- | --- | --- | --- | --- | --- | --- | --- | --- |
|  | Pilot Data gathering | **X** |  |  |  |  |  |  |  |  |  |  |  |  |  |  |  |  |  |  |  |  |  |  |  |  |  |  |  |  |  |
|  | Recruitment | **X** |  |  |  |  |  |  |  |  |  |  |  |  |  |  |  |  |  |  |  |  |  |  |  |  |  |  |  |  |  |
| 1 | Final data gathering |  | **X** | **X** | **X** | **X** | **X** |  |  |  |  |  |  |  |  |  |  |  |  |  |  |  |  |  |  |  |  |  |  |  |  |
| 2 | Statistical analysis |  |  | **X** |  |  |  | **X** |  |  |  |  |  |  |  |  |  |  |  |  |  |  |  |  |  |  |  |  |  |  |  |
| 3 | Manuscript writing |  |  |  |  |  |  | **X** | **X** |  |  |  |  |  |  |  |  |  |  |  |  |  |  |  |  |  |  |  |  |  |  |

| **9- Ethics:**   - Conscious consent is obtained from all patients. - Study procedure, patient’s role and the possibility of placing him/ her in intervention or control group will be fully clarified for the patient. - The research conduction won’t cause any harm or cost for the patients. - The patient is free to leave the study with/without any reason at any stage which will not harm his/her treatment. - The protection of patients’ personal information and principle of confidentiality will be fully observed. - Due to the fact that the patient may not be conscious enough to understand participation matter, only patients who are fully conscious will be enrolled in this study. |
| --- |

| **12 - References:**  1. Zhu N, Zhang D, Wang W, Li X, Yang B, Song J, et al. A novel coronavirus from patients with pneumonia in China, 2019. New England Journal of Medicine. 2020.  2. Read JM, Bridgen JR, Cummings DA, Ho A, Jewell CP. Novel coronavirus 2019-nCoV: early estimation of epidemiological parameters and epidemic predictions. MedRxiv. 2020.  3. Huang C, Wang Y, Li X, Ren L, Zhao J, Hu Y, et al. Clinical features of patients infected with 2019 novel coronavirus in Wuhan, China. The lancet. 2020;395(10223):497-506.  4. Zhou M, Zhang X, Qu J. Coronavirus disease 2019 (COVID-19): a clinical update. Frontiers of medicine. 2020:1-10.  5. Chen J. Pathogenicity and transmissibility of 2019-nCoV—a quick overview and comparison with other emerging viruses. Microbes and infection. 2020.  6. Liu T, Hu J, Kang M, Lin L, Zhong H, Xiao J, et al. Transmission dynamics of 2019 novel coronavirus (2019-nCoV). 2020.  7. Li Q, Guan X, Wu P, Wang X, Zhou L, Tong Y, et al. Early transmission dynamics in Wuhan, China, of novel coronavirus–infected pneumonia. New England Journal of Medicine. 2020.  Tang CY, Taylor NF, Blackstock FC. Chest physiotherapy for patients admitted to hospital with an acute exacerbation of chronic obstructive pulmonary disease (COPD): a systematic review. Physiotherapy. 2010 Mar 1;96(1):1-3.  9. Wujtewicz M, Dylczyk-Sommer A, Aszkiełowicz A, Zdanowski S, Piwowarczyk S, Owczuk R. COVID-19–what should anaethesiologists and intensivists know about it? Anaesthesiology intensive therapy. 2020;52(1):34-41.  10. Xu Z, Shi L, Wang Y, Zhang J, Huang L, Zhang C, et al. Pathological findings of COVID-19 associated with acute respiratory distress syndrome. The Lancet respiratory medicine. 2020;8(4):420-2.  11. Franks TJ, Chong PY, Chui P, Galvin JR, Lourens RM, Reid AH, et al. Lung pathology of severe acute respiratory syndrome (SARS): a study of 8 autopsy cases from Singapore. Human pathology. 2003;34(8):743-8.  12. Zhang H, Kang Z, Gong H, Xu D, Wang J, Li Z, et al. The digestive system is a potential route of 2019-nCov infection: a bioinformatics analysis based on single-cell transcriptomes. BioRxiv. 2020.  13. Baig AM. Computing the Effects of SARS-CoV-2 on Respiration Regulatory Mechanisms in COVID-19. ACS Chemical Neuroscience. 2020.  14. . !!! INVALID CITATION !!! (12, 13).  15. Mirastschijski U, Dembinski R, Maedler K. Lung Surfactant for Pulmonary Barrier Restoration in Patients With COVID-19 Pneumonia. Frontiers in Medicine. 2020;7:254.  16. Jin X, Lian J-S, Hu J-H, Gao J, Zheng L, Zhang Y-M, et al. Epidemiological, clinical and virological characteristics of 74 cases of coronavirus-infected disease 2019 (COVID-19) with gastrointestinal symptoms. Gut. 2020;69(6):1002-9.  17. Thomas P, Baldwin C, Bissett B, Boden I, Gosselink R, Granger CL, et al. Physiotherapy management for COVID-19 in the acute hospital setting: clinical practice recommendations. Journal of Physiotherapy. 2020.  18. Liu K, Zhang W, Yang Y, Zhang J, Li Y, Chen Y. Respiratory rehabilitation in elderly patients with COVID-19: A randomized controlled study. Complementary therapies in clinical practice. 2020:101166.  19. Ambrosino N, Foglio K, Rubini F, Clini E, Nava S, Vitacca M. Non-invasive mechanical ventilation in acute respiratory failure due to chronic obstructive pulmonary disease: correlates for success. Thorax. 1995;50(7):755-7.  20. Munshi L, Kobayashi T, DeBacker J, Doobay R, Telesnicki T, Lo V, et al. Intensive care physiotherapy during extracorporeal membrane oxygenation for acute respiratory distress syndrome. Annals of the American Thoracic Society. 2017;14(2):246-53.  21. Yang F, Liu N, Hu J, Wu L, Su G, Zhong N, et al. Pulmonary rehabilitation guidelines in the principle of 4S for patients infected with 2019 novel coronavirus (2019-nCoV). Zhonghua jie he he hu xi za zhi= Zhonghua jiehe he huxi zazhi= Chinese journal of tuberculosis and respiratory diseases. 2020;43(3):180-2.  22. Wang TJ, Chau B, Lui M, Lam G-T, Lin N, Humbert S. PM&R and Pulmonary Rehabilitation for COVID-19. American Journal of Physical Medicine & Rehabilitation. 2020.  23. Iannaccone S, Castellazzi P, Tettamanti A, Houdayer E, Brugliera L, de Blasio F, et al. Role of Rehabilitation Department for Adult Individuals With COVID-19: The Experience of the San Raffaele Hospital of Milan. Archives of Physical Medicine and Rehabilitation. 2020.  24. Lazzeri M, Lanza A, Bellini R, Bellofiore A, Cecchetto S, Colombo A, et al. Respiratory physiotherapy in patients with COVID-19 infection in acute setting: a Position Paper of the Italian Association of Respiratory Physiotherapists (ARIR). Monaldi Archives for Chest Disease. 2020;90(1).  25. Yang L-L, Yang T. Pulmonary rehabilitation for patients with coronavirus disease 2019 (COVID-19). Chronic Diseases and Translational Medicine. 2020.  26. Simpson R, Robinson L. Rehabilitation After Critical Illness in People With COVID-19 Infection. American journal of physical medicine & rehabilitation. 2020;99(6):470.  27. Hillegass E. Essentials of Cardiopulmonary Physical Therapy-E-Book: Elsevier Health Sciences; 2016.  28. of the International CSG. The species Severe acute respiratory syndrome-related coronavirus: classifying 2019-nCoV and naming it SARS-CoV-2. Nature Microbiology. 2020;5(4):536.  29. Theodore AC. Venous blood gases and other alternatives to arterial blood gases. Up to Date. 2019.  30. Ak A, Ogun CO, Bayir A, Kayis SA, Koylu R. Prediction of arterial blood gas values from venous blood gas values in patients with acute exacerbation of chronic obstructive pulmonary disease. The Tohoku journal of experimental medicine. 2006;210(4):285-90.  31. Bohannon RW, Bubela DJ, Wang Y-C, Magasi SS, Gershon RC. Six-minute walk test versus three-minute step test for measuring functional endurance (Alternative Measures of Functional Endurance). Journal of strength and conditioning research/National Strength & Conditioning Association. 2015;29(11):3240.  32. Durand F. How to improve long‐term outcome after liver transplantation? Liver International. 2018;38:134-8.  33. Hafen BB, Sharma S. Oxygen saturation. StatPearls [Internet]: StatPearls Publishing; 2019.  34. Efird J. Blocked randomization with randomly selected block sizes. International journal of environmental research and public health. 2011;8(1):15-20.  35. Doig GS, Simpson F. Randomization and allocation concealment: a practical guide for researchers. Journal of critical care. 2005;20(2):187-91.  36. Borg GA. Psychophysical bases of perceived exertion. Medicine & Science in Sports & Exercise. 1982.  37. Moher D, Liberati A, Tetzlaff J, Altman DG, Group P. Preferred reporting items for systematic reviews and meta-analyses: the PRISMA statement. PLoS med. 2009;6(7):e1000097. |
| --- |
