# Supplementary material for: Safety and efficacy of pulmonary physiotherapy in hospitalized patients with severe COVID-19 pneumonia (PPTCOVID study): A prospective, randomised, single-blind, controlled trial
Source: PLoS One. 2023 Jan 31;18(1):e0268428. doi: 10.1371/journal.pone.0268428 (PMC9888698; doi:10.1371/journal.pone.0268428)
Supplement: S2 Text — (DOCX) [file pone.0268428.s004.docx]

**فرم مخصوص ثبت پروپوزال کارشناسی ارشد و دکتری تخصصی**

1- **اطلاعات مربوط به دانشجو**

| نام و نام خانوادگي: محمد جواهریان شماره دانشجويي: 9611342001 |
| --- |
| رشته تحصيلي: فیزیوتراپی مقطع تحصيلي: دکتری تخصصی (PhD) |
| آدرس: تهران – گیشا – خیابان فروزانفر – پلاک 8 – واحد 5 تلفن: 88284847-021 |
| آدرس پست الکترونیکی: Javaherian_m@razi.tums.ac.ir تلفن همراه: 09129321391 |

2- **عنوان پايان‌نامه/رساله**

| **فارسی:**  " بررسی تاثیر فیزیوتراپی تنفسی بر روی یافته های بالینی بیماران بستری مبتلا به پنومونی ناشی از کوروناویروس جدید 2019 " |
| --- |
| **English:**  “Efficacy of Chest Physiotherapy on Hospitalized Patients with Novel coronavirus 2019 Pneumonia” |

**واژه های کلیدی**

| **فارسی:**  فیزیوتراپی تنفسی – کوروناویروس جدید 2019 – کووید-19 – پنومونی |
| --- |
| **English:**  Pulmonary Physiotherapy– Novel Coronavirus 2019 – COVID-19 – Pneumonia |

**3- مشخصات استاد راهنمای اول و دوم**

| نام‌خانوادگي: عطارباشی مقدم نام: بهروز تخصص اصلی: فیزیوتراپی |
| --- |
| آخرين مدرك تحصيلي و رتبه دانشگاهی: دکتری تخصصی - دانشیار ایمیل:Attarbashi@tums.ac.it |
| آدرس: تهران – خیابان انقلاب – پیچ شمیران – دانشکده توانبخشی دانشگاه علوم پزشکی تهران – گروه فیزیوتراپی تلفن: 0912183095 |

4- مشخصات استاد مشاور اول

| نام‌خانوادگي: شادمهر نام: آزاده تخصص اصلی: فیزیوتراپی |
| --- |
| آخرين مدرك تحصيلي و رتبه دانشگاهی: دکتری تخصصی - استاد ایمیل:Shadmehr@tums.ac.ir |
| آدرس: تهران – خیابان انقلاب – پیچ شمیران – دانشکده توانبخشی دانشگاه علوم پزشکی تهران – گروه فیزیوتراپی  تلفن: 09123703379 |

**5- مشخصات استاد مشاور دوم**

| نام‌خانوادگي: بیگ محمدی نام: محمدتقی تخصص اصلی: بیهوشی – فلوشیپ مراقب های ویژه |
| --- |
| آخرين مدرك تحصيلي و رتبه دانشگاهی: فلوشیپ - دانشیار ایمیل: mbage46@gmail.com |
| آدرس: تهران – انتهای بلوار کشاورز – مجمتع بیمارستانی امام خمینی – ساختمان انستیتوکانسر– بخش مراقبت های ویژه کانسر  تلفن: 09121759016 |

6- **جزئيات طرح پايان نامه**

**1-5. مقدمه و بیان مسئله**

کرونا ویروس جدید از اواسط دسامبر ۲۰۱۹ در شهر ووهان چین پدیدار و به عنوان علت پنومونی در تعداد زیادی از بیماران چینی تایید شد (1). این ویروس که کووید -۱۹ نام گذاری شده است، هم اکنون شرایط پاندمیک به خود گرفته و تاکنون در تمام کشور دنیا گسترش یافته و بیش از 16 میلیون نفر را مبتلا کرده است. بنا بر گزارشات رسمی از جانب وزارت بهداشت، درمان و آموزش پزشکی شیوع این بیماری از ۲۹ بهمن ۱۳۹۸ در ایران نیز تایید شده است. این بیماری معمولا از طریق استنشاق ذرات معلق آلوده به ویروس منتقل می شود. دوره ی کمون بیماری تقریبا ۳ تا ۷ روز گزارش شده است و معمولا بیشتر از ۱۴ روز ادامه نمی یابد(2). شایع ترین نشانه ها و علائم کووید -۱۹ شامل تب، سرفه خشک، تنگی نفس و خستگی می باشد. تعدادی از بیماران علائمی مانند احتقان بینی، آبریزش بینی و اسهال نیز دارند. در موارد شدیدتر، این عفونت می تواند باعث ایجاد پنومونی، سندروم حاد تنفسی شدید و گاهی مرگ شود (3). در مطالعاتی نیز گزارش شده است که عوراض ناشی از کووید-19 می تواند طولانی باشد (4).

بر اساس شواهد موجود بیماری زایی کووید -۱۹حدود سه درصد است (5). این ویروس از قابلیت انتقال بالایی برخوردار است به طوری که هر عفونت می تواند به ۲/۲ یا ۹/۲ فرد دیگر منتقل شود (6, 7). از این رو به نظر می رسد اعمال مداخلات درمانی به موقع در مدیریت شیوع و گسترش کروناویروس جدید از اهمیت قابل ملاحظه ای برخوردار است.

فیزیوتراپی تنفسی یک متد درمانی ساده ، جامع، مؤثر ، ایمن و بی خطر است که با هدف بهبود علائم تنفسی بیماران از طریق آموزش سرفه موثر، تخلیه ی ترشحات راه هوایی، تمرینات تنفسی و از بین بردن عوامل تشدید کننده و غیره می تواند در مدیریت بیماری های تنفسی موثر واقع شود و با بهبود شرایط بیمار در هزینه های درمانی صرفه جویی نماید. مطالعات سابق در زمینه اثرگذاری فیزیوتراپی در بیماران مبتلا به اختلالات گوناگون تنفسی از جمله آسم و بیماری مزمن انسدادی ریه نشان می دهد که این تکنیک ها می توانند موجب افزایش فعالیت فیزیکی، افزایش سطح کیفیت زندگی و ظرفیت هوازی بیمار شود (8).

بیماران مبتلا به کووید-19 به دلیل طول درمان طویل خود در بخش های پیش مراقبت ویژه و مراقبت ویژه غالبا دچار اختلالات فیزیکی، روانی و ریوی می شوند. از طرفی این بیماران مجبور هستند که بعد از زمان ترخیص خود در محیط قرنطینه باقی مانده که خود این امر می­تواند موجب افزایش اختلالات فوق می شود. از منظر تئوری انتظار می رود که این ویروس بتوانند تا مدت ها چنین اختلالی را برای بیماران ایجاد کرده و موجب محدودیت فعالیت های فیزیکی آن ها شود (9). در یک مطالعه کوهورت با 109 نمونه نجات یافته از سندروم حاد تنفسی نشان داده شد که حجم ریه و معیارهای اسپیرومتری بیماران 6 ماه پس از ابتلا کاملا نرمال بود ولی ظرفیت تبادل دی اکسید کربن ریه و ظرفیت هوازی آن ها کماکان مختل بود. این موضوع نشان دهنده این است که پنومونی ویروسی می تواند ایجاد بافت شبیه فیبروتیک در سطح ریه کند (10).

تاکنون پژوهشگران مختلفی بر روی مکانیسم ایجاد پنومونی ناشی از کوروناویروس جدید 2019 مطالعه کرده اند ولی بین آن ها نتیجه واحده­ای در ارتباط با این مکانیسم وجود ندارد. یکی از مکانیسم های نسبتا قوی که درحال حاضر پژوهشگران علوم دارویی نیز از آن برای پژوهش های خود استفاده می کنند، توسط Franks و همکاران در ارتباط SARS-CoV در سال 2003 منتشر شد (11). بر طبق یافته های آن ها سلول هدف کوروناویروس در بافت ریه، نوموسیت نوع II بوده که مسئول تولید سورفاکتانت و زمینه ساز ایجاد سلول های نوموسیت نوع I می باشد. از طرفی بر طبق یافته های Zhang و همکاران گیرنده های ACE2^[[1]](#footnote-1)^ اصلی ترین گیرنده SARS-CoV-2 بوده که در سطح سلول نوموسیت نوع II بوفور وجود دارد (12). بنابراین کاهش ترشح سورفاکتانت و سایر پروتوگلیکان ها می تواند باعث افزایش میزان سفتی بافت آلوئولی، تمایل آن ها به کلاپس و نهایتا کاهش سطح پرفیوژن گازهای تنفسی شود. بنابراین پنومونی ناشی از کووید-19 بعنوان یک پنومونی ویروسی می تواند موجب تغییرات بیومکانیکی (بخصوص خواص ویسکوالاستیک) بافت آلوئولی و Intersitial Space شود (9). این یافته ها کاملا با نظر Baig سازگار است که بیان می کند مهمترین دلیل ایجاد پنومونی ناشی از کووید-19 را کاهش پرفیوژن ریوی و Vasoconstriction هایپوکسیک می داند (13).

با توجه به اینکه در پنومونی ناشی از کوروناویروس جدید فیلتراسیون اکسیژن و دی اکسید کربن در لایه اندوتلیوم مختل می شود، به نظر می رسد اکسیژن درمانی و روش هایی همانند استفاده از تهویه مکانیکی از طرق مختلف (Continuous Positive Airway Pressure (CPAP)^[[2]](#footnote-2)^, Bilevel Positive Airway Pressure (BiPAP) و غیره)، روش های درمانی غیردارویی مناسبی برای این بیماری باشد. از آنجایی تکنیک های متعددی در روش های فیزیوتراپی تنفسی وجود دارد که می تواند منجر به تهویه بهتر در سطح آلوئول گردد، بنابراین بنظر می رسد این روش ها می توانند در مدیریت این اختلال یک روش درمانی اثرگذار باشد (14). فرضیه بیومکانیکی این روش ها نیز استفاده از خاصیت Creep و سایر خواص وابسته به زمان مواد ویسکوالاستیک می باشد که در طی آن بتوان سفتی نسبی بافت آلوئولی را که به دلیل عدم ترشح کافی ماده سورفاکتانت بوجود می آید، کاهش داد (15). از طرفی بر طبق گزارش های موجود ۳۴ درصد از بیماران مبتلا به پنومونی ناشی از کووید-19 دارای ترشحات ریوی قابل سمع می باشند (16) و محتمل است اعمال مداخلات فیزیوتراپی تنفسی جهت تخلیه ترشحات ریوی بتواند موجب ارتقای علائم بالینی بیمار شود.

در حال حاضر سطوح شواهد علمی در ارتباط با اثرگذاری روش درمانی فیزیوتراپی تنفسی در فاز بستری در سطحی است که بیشتر متمرکز بر یافته های محدود قبلی در مواجهه با Middle-East Respiratory Syndrome (MERS) یا Sever Acute Respiratory Syndrome (SARS) و تجربیات بالینی و نظر افراد ماهر در این زمینه است. این سطوح شواهد به اندازه ای هست که بتوان فیزیوتراپی تنفسی را از منظر تئوری یک روش موثر درمانی تلقی کرد ولی به اندازه ای کافی نیست که بتوان از نظر بالینی اثرگذاری آن را برای تمام بیماران تائید کرد.

از طرفی، از چندماه گذشته که فیزیوتراپیست های جهان با پاندمی کورونا مواجه بودنده اند، دو سوال اصلی ذهن آن ها در ارتباط با تجویز فیزیوتراپی برای بیماران درگیر کرده است. اول اینکه آیا فیزیوتراپی تنفسی در شرایط حاد بیماری می تواند موثر باشد؟ گروه دیگری نیز این بحث را مطرح می کردند که نقش فیزیوتراپی در شرایط حاد صرفا برای بیماران دارای ترشحات اضافی ریوی است. گایدلاین منتشر شده از جانب کنفدراسیون جهانی فیزیوتراپی و توصیه های منتشر شده از جانب پژوهشگران استرالیایی شاهدی بر وجود چنین نگرشیی است (17). اما زمانی که به مکانیسم ایجاد پنومونی ناشی از کووید-19 و بخصوص بحث کاهش ترشح سورفاکتانت توجه می کنیم، به یک اختلال بیومکانیکی میرسیم که این ایده را در ذهن ایجاد می کند که شاید آن دسته از تمرینات تنفسی که منجر به کاهش سفتی بافت آلوئول می شوند، بتوانند در بهبود علائم و شاید درمان بیماران مبتلا به کووید-19 موثر باشد. فرضیه ما ناقض اثرگذاری احتمالی تکنیک های تخلیه ترشحات تنفسی در آن دسته از بیمارانی که دارای ترشحات هستند، نیست ولی بیان می کند که ممکن است هم بیماران با و هم بیماران بدون ترشحات اضافی ریوی بتوانند تکنیک های تنفسی در راستای کاهش سفتی بافت آلوئولی بهره مند شوند. بر طبق این فرضیه بیماران مبتلا به پنومونی ناشی از کوروناویروس را باید به دو گروه تقسیم کرد؛ نخست گروهی که دارای ترشحات اضافی ریوی هستند که این بیماران بایستی در ابتدا تکنیک های تخلیه ترشحات تنفسی و سپس تکنیک های تنفسی (همانند کنترل دیافراگماتیک، حبس تنفس ویا کنترل تنفسی) را دریافت کنند و گروه دوم بیمارانی هستند که معاینه بالینی آن ها وجود تکنیک های تخلیه ترشحات ریوی را ثابت نمی کند. برای این دسته از بیماران بایستی صرفا تکنیک های کنترل تنفسی استفاده کرد.

در یک کارآزمایی بالینی اخیرا منتشر شده با 72 حجم نمونه بیماران مسن ترخیص شده از بیمارستان به دلیل کووید-19 نشان داده شد که 12 جلسه فیزیوتراپی کوتاه مدت (به مدت 6 هفته) می تواند میزان اضطراب، افسردگی و سطح ناتوانی بیمار را تا حد قابل توجهی کاهش داده و سطح کیفیت زندگی و عملکرد هوازی آن ها را افزایش دهد (18). این مطالعه تنها کارآزمایی بالینی منتشر شده در زمینه فیزیوتراپی تنفسی در فاز پس از ترخیص بیماران مبتلا به کووید-19 است.

بنابراین ما در حال حاضر با یک خلا محسوس علمی مواجه هستیم که می توان آن را با یک سلسله مطالعات پژوهشی در قالب کارآزمایی بالینی تصادفی کنترل شده پر کرد تا بتوان با اطمینان بیشتری فیزیوتراپی تنفسی را برای تمام بیماران تجویز کرد. هدف این مطالعه کارآزمایی بالینی تصادفی کنترل شده بررسی اثرگذاری برنامه فیزیوتراپی تنفسی در فاز بستری بیماران مبتلا به کووید-19 بر یافته های تنفسی، کیفیت زندگی، بروز مرگ و میر و بستری مجدد می باشد.

**2-5. جنبة جديد بودن موضوع**

با توجه به شیوع گسترده ویروس SARS-CoV-2^[[3]](#footnote-3)^ در سرتاسر جهان و ایجاد پنومونی گسترده در سطح جامعه، بنظر می رسد نیاز است در قالب یک مطالعه کارآزمایی بالینی کنترل شده اثرات درمانی کوتاه مدت و بلند مدت مداخله فیزیوتراپی تنفسی در فاز بستری بررسی شود. از طرفی از آنجایی در حال حاضر بین متخصصان فیزیوتراپی سوال وجود دارد که این درمان را برای تمام بیماران ویا صرفا برای بیماران دارای ترشحات ریوی استفاده کنیم، هدف دوم ما این است که نتایج درمانی حاصل شده را در بین دو گروه بیماران با و بدون ترشحات تنفسی مقایسه کنیم. نتایج این پژوهش با سایر گزارشات این حوزه مقایسه خواهد شد و در قالب یک پنل خبرگان این حوزه با جزئیات بررسی می شود. با توجه به نظرات خبرگان تلاش می شود پروتکل درمانی فیزیوتراپی تنفسی در فاز بستری در بیماران مبتلا به پنومونی ناشی از کوروناویروس جدید 2019 تدوین گردد.

**3-5. سابقه علمی پژوهش های انجام شده**

AMBROSINO و همکارانش در سال 2013 به برسی تکنیک های فیزیوتراپی در درمان بیماران سندرم حاد تنفسی (ARDS^[[4]](#footnote-4)^) پرداختند. در این مطالعه از روش های تمرین درمانی، Mobilization، تحریک الکتریکی، Manual hyperinflation ،Percussion و In-ex sufflation^[[5]](#footnote-5)^ استفاده شد. نتایج به دست آمده نشان داد که در بیماران ARDS در که بخش مراقبت های ویژه بستری هستند، مداخلات فیزیوتراپی باید در کوتاهترین زمان ممکن شروع شود(1۴). بعبارتی دیگر اثرگذاری روش های درمانی فیزیوتراپی می تواند ثانویه به زمان آغاز آن باشد (19).

Munshi و همکارانش در سال ۲۰۱۷، در یک مطالعه ی کوهورت گذشته نگر به بررسی تاثیرات مداخلات فیزیوتراپی در بخش مراقبت های ویژه در زمان استفاده از Extracorporeal Membrane Oxygenation (ECMO) برای بیماران سندرم حاد تنفسی ARDS پرداختند. در این مطالعه، میزان سطح فعالیت روزانه این بیماران از طریق مقیاس ICU Mobility Scale کدگذاری شد. ۶۱ نفر از ۱۰۷ مریض وابسته به ECMO دچار سندرم حاد تنفسی بودند و فیزیوتراپی در بخش مراقبت های ویژه برای ۸۲ درصد آنان انجام شد. با توجه به نتایج به دست آمده، فیزیوتراپی در طول ECMO در صورتی که از طرف تیم متخصص انجام بگیرد کاملا ایمن بوده و قابل انجام می باشد (20).

Yang و همکارانش در سال 2020 مطالعه ای بر روی بیماران مبتلا به عفونت ریه که به ویروس کووید -۱۹ آلوده شده بودند، انجام دادند. در این مطالعه به بررسی تاثیرات توانبخشی ریوی به منظور جلوگیری از شیوع ویروس، هدایت بیمار برای انجام شرکت در برنامه ی توانبخشی ریوی و انجام تمرینات عضلات تنفسی، دفع ترشحات و بهبود سلامت روان بیماران پرداخته شد. تمرینات تنفسی، اموزش سرفه موثر، تمرین مقاومتی برای تقویت عضلات تنفسی، تمرینات کششی اندام ها، تمرینات تقویتی برای اندام فوقانی و تحتانی، پل زدن، دوچرخه زدن در هوا توسط بیمار و تحت نظارت تراپیست انجام شد. تمرینات دو بار در روز و هر تمرین ۲۰-۱۵ بار برحسب تحمل بیمار تکرار شد. بیماران در صورت هیپوکسی در طول برنامه توانبخشی اکسیژن دریافت می کردند. بیماران دارای سرفه خلط دار،تمام تمرینات تنفسی را دو بار در روز با ۵۰ تکرار انجام دادند. شدت تمرینات برحسب میزان مقاومت اعمال شده توسط بیماران تنظیم می شد. با توجه به نتایج به دست آمده از این مطالعه، روش های درمانی فیزیوتراپی برای بیمار بی خطر است و از آن جایی که این درمان تحت تاثیر زمان، مکان و امکانات قرار نمی گیرد، فیزیوتراپیست می تواند تکنیک ها را در خانه یا در بخش مراقب های ویژه به راحتی اعمال کند. همچنین به دلیل بازده بالا سبب رضایت بیمار و پرسنل می شود. از نکات مهمی که در اعمال فیزیوتراپی این بیماران حائز اهمیت است، امکان انجام درمان آن از طریق ویدیو و تلفن از راه دور می باشد که در جلوگیری از گسترش بیماری از اهمیت بالایی برخوردار است (21). متاسفانه این مطالعه کاملا Opinion-based بوده و از طراحی مطالعات کمی تبعیت نکرده است.

در سال 2020، Wang و همکاران از کشور آمریکا در طی یک مطالعه مروری با بررسی شواهد قبلی در ارتباط با پنومونی های ویروسی، بیماری های SARS/MERS و تجربیات متخصصین مختلف حوزه توانبخشی، به مرور شواهد کنونی جهت مدیریت فیزیوتراپی بیماران مبتلا به کووید-19 پرداختند. در این مطالعه ، مهمترین هدف توانبخشی تنفسی بیماران بهبودی علائم تنگی تنفس، کاهش سطح استرس، کاهش مشکلات ناشی از عفونت، کاهش سطح ناتوانی و حفظ ظرفیت عملکردی و کیفیت زندگی بیماران معرفی شدند. در این مطالعه تکنیک های توانبخشی ریوی بیماران شامل تکنیک های تخلیه ترشحات تنفسی، اصلاح پاسچر، تمرینات تنفسی و مخصوصا تنفس دیافراگماتیک، تمرینات کششی، درمان های دستی و تمرینات هوازی مرور شدند. بر طبق نظر این محققین اقدامات فیزیوتراپی تنفسی می توانند از شرایط حاد شروع شده و تا بازه زمانی پس از ترخیص ادامه داشته باشد (22). علی رغم اجرای بسیار مناسب روش جستجوی مقالات اولیه، پژوهشگران این مقاله نتوانستند نهایتا یک مطالعه مرتبط با اثرات روش فیزیوتراپی تنفسی در فاز بستری بر روی بیماران مبتلا به SARS/MERS بیابند.

Iannaccone و همکاران از کشور ایتالیا در سال 2020 با انتشار یک مقاله نظریه محور به گزارشی از شرایط بیمارستان San Raffaele شهر میلان و بیان تجربیات خود در ارتباط با توانبخشی بزرگسالان مبتلا به کووید-19 پرداختند. بر طبق آمار این پژوهشگران مدت زمان اقامت بیماران کووید-19 در این بیمارستان 15 روز بوده است. از آنجایی این زمان طولانی می تواند موجب کاهش چشمگیر سطح توانایی فیزیکی و روانی بیمار شود و تیم توانبخشی این بیمارستان یافتند که حدود 20% از بیماران حائز بستری شدن در بخش های توانبخشی هستند، مسئولان بخش توانبخشی تصمیم گرفتند که بیماران پس از بستری در بخش های حاد وارد بخش های توانبخشی ویا قرنطینه شوند. بیماران پس از بستری در این بخش ها تا زمانی که بتوانند فعالیت های عملکردی خود را بازیابند، در بخش های توانبخشی بستری شدند. هدف برنامه توانبخشی بیماران کووید-19 در بخش های حاد و پس از حاد این بیمارستان، افزایش سطح دینامیک تنفسی، مقابله با اختلال سیستم عضلانی-اسکلتی، کاهش بروز مشکلات بستری، احیای شرایط شناختی و روحی بیماران، کاهش سطح ناتوانی و ارتقای کیفیت زندگی بیماران در زمان پس از ترخیص بیماران است (23).

مداخلات فیزیوتراپی در فاز بستری بیماران شامل 1) مدیریت پاسچر، 2) مدیریت بین رشته¬ای ونتیلاسیون غیر تهاجمی^[[6]](#footnote-6)^، 3) احیای حرکات فعال و غیر فعال بیمار و 4) تعیین معیارهای تنفسی و حرکتی بیمار جهت ترخیص بودند. همچنین بیماران بستری در بخش های توانبخشی مداخلات تمرینات هوازی، مقاومتی، تعادلی، بهبود فعالیت های روزانه و بهبود شرایط شناختی (توسط روانشناس) دریافت می کردند. نتیجه تمام این اقدامات انجام شده کاهش مدت زمان بستری در بیمارستان به 10 روز بود (23).

همچنین تمام این بیماران پس از ترخیص از قرنطینه یا بخش های توانبخشی، از طریق سیستم توانبخشی از راه دور تحت نظارت تیم توانبخشی بودند.

در ماه مارچ 2020، Lazzeri و همکاران از جانب انجمن فیزیوتراپی تنفسی ایتالیا یک Position Paper منتشر کردند که در آن جوانب مختلف فیزیوتراپی تنفسی در بیماران مبتلا به کووید-19 از جمله احتمال انتقال این ویروس، انواع روش های تهویه بیمار، تغییر پاسچر بیمار، تکنیک های تخلیه ترشحات تنفسی، نحوه کار با سیستم ونتیلاتور، معیارهای Weaning و مشکلات ممکن رخ داده در بیماران بستری در ICU پرداختند (24).

Liu و همکاران از کشور چین در ماه مارچ 2020 با انتشار یک مطالعه کارآزمایی بالینی تصادفی همراه با گروه کنترل به بررسی اثرات درمانی 6 هفته توانبخشی تنفسی در بروی عملکرد ریوی (از طریق اسپیرومتری)، سطح کیفیت زندگی (بوسیله پرسشنامه SF-36)، ظرفیت هوازی (از طریق تست 6 دقیقه راه رفتن)، میزان فعالیت فیزیکی (از طریق معیار استقلال عملکردی) و عملکرد روانی (بوسیله خوداظهاری سطح افسردگی و اضطراب) در بیماران مسن (با سن بیشتر از 65 سال) که از بیمارستان ترخیص شده بودند، پرداختند. بیماران گروه مداخله به مدت 6 هفته تحت 12 جلسه توانبخشی به مدت 10 دقیقه با تمرکز بر تمرینات تنفسی، سرفه موثر، تمرینات هوازی و مقاومتی و کششی قرار گرفتند. نتایج این مطالعه نشان داد که این برنامه توانبخشی می تواند موجب بهبودی معنی دار آماری در مقایسه با گروه کنترل در تمام معیارهای مذکور شود (18).

در می سال 2020، Lu-Lu Yang و Ting Yang از کشور چین با انتشار یک مطالعه مروری به بررسی انواع مداخلات ممکن توانبخشی جهت بیماران بستری و ترخیص شده مبتلا به کووید-19 پرداختند. بر طبق یافته های این مطالعه سه تست ارزیابی Breath-hold test، 1-minute step test و Squat بعنوان تست های مطرح برای بیماران مبتلا به کووید-19 مطرح شده اند. بر طبق دیدگاه این پژوهشگران تکنیک های توصیه شده برای این بیماران شامل تنفس دیافراگماتیک (در طی 10 دقیقه با فرکانس 3 بار در روز)، تکنیک های تخلیه ترشحات ریوی (شامل تمرینات بازدمی در طی 10 دقیقه و فرکانس 1 بار در روز)، تمرینات عضلات تنفسی، تمرینات Expansion سینه ای (شامل کشش عضلات به مدت 5 دقیقه و با فرکانس 1 بار در روز)، تمرینات هوازی (به مدت 30-10 دقیقه راه رفتن با ضربان قلب هدف 124 ضربان در دقیقه، میزان خستگی بیشتر از 2 بر مبنای مقیاس Borg و میزان Saturation اکسیژن خون بیشتر از 90 درصد) و تمرینات مقاومتی (با استفاده از باند الاستیک یا وزنه) می باشند. اینکه آیا این تمرینات باید برای تمام بیماران و با هر شرایطی استفاده شود یا اینکه بایستی انتخابی باشد، سوالی است که در این مطالعه به آن پاسخ داده نشده است (25).

در سال 2020، Thomas و همکاران با انتشار یک مقاله Clinical Practice Recommendation، به مروری بر تکنیک های مختلف فیزیوتراپی برای بیماران مبتلا به کووید-19 پرداختند. این مطالعه به جنبه های مختلف فیزیوتراپی بیماران کووید-19 از جمله تعداد نیروی فیزیوتراپیست مورد نیاز، تجهیزات فیزیوتراپی مورد نیاز در بخش های کووید-19، احتیاطات مراقبتی کلینیسین های فیزیوتراپی و تکنیک های فیزیوتراپی تنفسی پرداختند. نکته جالب این مطالعه این است که نویسندگان آن همانند گایدلاین منتشر شده از سوی کنفدراسیون جهانی فیزیوتراپی غالب تکنیک های خود را معطوف به اقدامات تخلیه ترشحات ریوی، استفاده از تهویه غیر تهاجمی فشار مثبت، تمرینات راه اندازی بیمار، اقدامات حین تهویه تنفسی مکانیکی در بخش مراقبت ویژه و تمرینات فعال و غیر فعال کرده اند و همانند نظر سایر محققین تمرینات هوازی و تمرینات Expansion ریوی را مطرح نکرده اند(17) .

Robinson و Simpson از کشور کانادا با انتشار یک مقاله Rapid literature review به بررسی وضعیت پاندمی کووید-19 در جهان و مروری بر آخرین اهداف سیستم جهانی توانبخشی برای این بیماران پرداختند. بر طبق یافته های آن ها سطح بالای اختلالات فیزیکی، شناختی و روانی در بیماران مبتلا به کووید-19 قابل انتظار بوده و سیستم توانبخشی می تواند نقش بسزایی در روند مراقبت و بازگشت به زندگی طبیعی بیماران مبتلا به کووید-19 داشته باشد. همچنین این برنامه توانبخشی می تواند نقش زیادی در کمک به حفظ ساختار اداری و اقتصاد جهان داشته باشد. همچنین آن ها اشاره­ای به نقش برنامه توانبخشی مجازی برای این گروه از بیماران داشتند (26).

با توجه به مرور مطالعات گذشته و ارزیابی دقیق آن ها در میابیم که تا بحال پژوهشگران حوزه فیزیوتراپی مطالعه ای در ارتباط با میزان اثرگذاری مداخلات فیزیوتراپی تنفسی در بیماران بستری به دلیل پنومونی ناشی از کووید-19 اجرا نکرده اند. تا کنون هر آنچه که داریم مبتنی بر نظر افراد ماهر در این زمینه بوده که بعضا با یکدیگر ضد و نقیض است. این مطالعه علاوه بر ارائه یک روش درمانی در بیماران فاز بستری مبتنی بر تصمیم گیری بالینی سعی در آن دارد که میزان اثرگذاری آن در قالب یک کارآزمایی بالینی تصادفی کنترل شده ارزیابی کند.

**4-5. تعریف واژه ها و مفاهیم**

**فیزیوتراپی تنفسی:**

تعریف شرحی: فیزیوتراپی تنفسی یک متد درمانی ساده ، جامع، مؤثر ، ایمن و بی خطر است که با هدف بهبود علائم تنفسی بیماران از طریق آموزش سرفه موثر، تخلیه ی ترشحات راه هوایی، تمرینات تنفسی و از بین بردن عوامل تشدید کننده و غیره می تواند در مدیریت بیماری های تنفسی موثر واقع شود و با بهبود شرایط بیمار در هزینه های درمانی صرفه جویی نماید (27).

تعریف عملکردی: در این پژوهش روش های مختلف فیزیوتراپی تنفسی در بیماران مبتلا به پنومونی ناشی از کوروناویروس جدید 2019 مبتنی بر تصمیم گیری بالینی در دو گروه تمرینات تهویه تنفسی و تکنیک های خروج ترشحات اضافی ریوی ارائه خواهد شد.

**کوروناویروس جدید 2019:**

تعریف شرحی: ویروس SARS-CoV2 یک ویروس از خانواده کوروناویروس ها بوده که موجب ایجاد بیماری کوروناویروس می شود. این بیماری می تواند خود را در قالب اختلالات تنفسی بروز دهد (28). این اختالات بصورت کلی پنومونی (عفونت ریوی) ریه

تعریف عملکردی: بیماران پژوهش حاضر افراد مبتلا به پنومونی ناشی از کوروناویروس بوده که در دسته بندی بیماران پیش مراقبت ویژه قرار می گیرند. ویژگی های این بیماران در قالب معیارهای ورود و خروج روش اجرا اشاره شده است.

**آنالیز گاز وریدی^[[7]](#footnote-7)^:**

تعریف شرحی: آنالیز گاز وریدی یک روش جایگزین جهت تخمین گازهای خونی (اکسیژن و دی اکسید کربن)، میزان اسیدیتی، درصد اشباع اکسیژن خونی و همچنین سطح بی کربنات خون است. در اصل این روش برای شرایطی کاربرد دارد که امکان آنالیز گازهای خونی شریانی وجود ندارد (29). بر طبق نتایج مطالعات قبلی اندازه های حاصل از آنالیز گاز وریدی در بیماران مبتلا به اختلالات سیستم تنفسی همبستگی بالا و معنی داری با اندازه های حاصل از آنالیز گاز شریانی دارد (30).

تعریف عملکردی: در این پژوهش از آنالیز گازهای وریدی جهت عملکرد ریوی قبل و بعد از دوره اعمال مداخلات استفاده خواهد شد. نمونه های VBG در محل بستری بیمار اخذ شده و بلافاصله به آزمایشگاه Arterial Blood Gasses بیمارستان امام خمینی ارسال خواهد شد. مدت زمان انتقال نمونه از بخش به آزمایشگاه کمتر از 10 دقیقه خواهد بود. بمنظور کنترل شرایط نمونه گیری، خونگیری از ورید های محیطی اندام فوقانی اخذ خواهد شد.

**تست 3 دقیقه راه رفتن:**

تعریف شرحی: تست 3 دقیقه راه رفتن یک تست ساده و بالینی بوده که پاسخ عمومی و جامع تمام سیستم های درگیر در ورزش را شامل سیستم قلبی-عروقی، سیستم ریوی، سیستم عضلانی-اسکلتی، سیستم عصبی-عضلانی و سیستم متابولیک بیان می کند (31).

تعریف عملکردی: در این پژوهش تست 3 دقیقه راه رفتن از تمام بیماران قبل و بعد از اعمالات مداخلات با درنظر گرفتن شرایط خاص آن ها گرفته خواهد شد.. معیار حاصله از این تست شامل میزان راه رفتن خواهد بود. همچنین از بیماران خواسته می شود که میزان خستگی احساس شده خود را در انتهای تست بر اساس مقیاس Borg (بین 6 تا 20) اعلام کنند.

**کیفیت زندگی:**

تعریف شرحی: بر طبق تعریف سازمان جهانی بهداشت، کیفیت زندگی بعنوان وضعیت کلی فیزیکی، روانی و اجتماعی فرد در نظر گرفته می شود که لزوما همراه با بیماری یا بدون آن نیست (32).

تعریف عملکردی: در این پژوهش کیفیت زندگی بیمار از طریق پرسشنامه Short form-36 که یک پرسشنامه کیفیت زندگی وابسته به سلامت است، اندازه گیری می شود.

**درصد اشباع اکسیژن خونی:**

تعریف شرحی: درصد اکسیژن خونی یک فاکتور حیاتی در مدیریت و درمان مراقبت از بیمار است. این درصد حاصل تعداد هموگلوبین های متصل شده به اکسیژن نسبت به کل هموگلوبین های خون است (33).

تعریف عملکردی: در این پژوهش درصد اشباع اکسیژن خونی قبل و بعد از اعمال مداخلات استفاده خواهد شد. برای اینکه ما شرایط کنترل شده داشته باشیم، این مقیاس دو دقیقه پس از استنشاق هوای آزاد و دو دقیقه استنشاق اکسیژن توسط Partial Rebreather یا ماسک رزرو با قرارگرفتن بیمار در یک وضعیت خاص اندازه گیری خواهد شد.

**5-5. هدف كلي**

هدف این مطالعه کارآزمایی بالینی تصادفی کنترل شده بررسی اثرگذاری برنامه فیزیوتراپی تنفسی در فاز بستری بیماران مبتلا به کووید-19 بر یافته های تنفسی، کیفیت زندگی، بروز مرگ و میر و بستری مجدد می باشد تا بتوان از طریق مرور شواهد قبلی، نتایج این کارآزمایی بالینی و نظر خبرگان حوزه بتوان به یک پروتکل درمانی فیزیوتراپی تنفسی در مدیریت بیماران بستری مبتلا به کووید-19 رسید

**6-5. سوالات و یا فرضیات پژوهش**

1. میانگین میزان فشار گازهای اکسیژن و دی کسید کربن و PH، بی کربنات و درصد اشباع اکسیژن خون وریدی در گروه های مداخله و کنترل به تفکیک قبل و بعد از مداخله فیزیوتراپی در بیماران مبتلا به کوروناویروس جدید 2019 چقدر است؟
2. میانگین میزان فشار گازهای اکسیژن و دی کسید کربن و PH، بی کربنات و درصد اشباع اکسیژن خون وریدی در بیماران مبتلا به کوروناویروس با و بدون ترشحات اضافی ریوی حاضر در گروه مداخله به تفکیک قبل و بعد از مداخله فیزیوتراپی چقدر است؟
3. میانگین درصد اشباع اکسیژن اکسیژن خونی پس از دو دقیقه استنشاق هوای آزاد در گروه های مداخله و کنترل به تفکیک قبل و بعد از مداخله فیزیوتراپی در بیماران مبتلا به کوروناویروس جدید 2019 چقدر است؟
4. میانگین درصد اشباع اکسیژن اکسیژن خونی پس از دو دقیقه استنشاق هوای آزاد در بیماران مبتلا به کوروناویروس با و بدون ترشحات اضافی ریوی حاضر در گروه مداخله به تفکیک قبل و بعد از مداخله فیزیوتراپی چقدر است؟
5. میانگین درصد اشباع اکسیژن اکسیژن خونی پس از دو دقیقه استنشاق اکسیژن توسط Partial Rebreather در گروه های مداخله و کنترل به تفکیک قبل و بعد از مداخله فیزیوتراپی در بیماران مبتلا به کوروناویروس جدید 2019 چقدر است؟
6. میانگین درصد اشباع اکسیژن اکسیژن خونی پس از دو دقیقه استنشاق اکسیژن توسط Partial Rebreather در بیماران مبتلا به کوروناویروس با و بدون ترشحات اضافی ریوی حاضر در گروه مداخله به تفکیک قبل و بعد از مداخله فیزیوتراپی چقدر است؟
7. میانگین میزان مسافت طی شده در تست 3 دقیقه راه رفتن در گروه های مداخله و کنترل به تفکیک قبل و بعد از مداخله فیزیوتراپی در بیماران مبتلا به کوروناویروس جدید 2019 چقدر است؟
8. میانگین میزان مسافت طی شده در تست 3 دقیقه راه رفتن در بیماران مبتلا به کوروناویروس با و بدون ترشحات اضافی ریوی حاضر در گروه مداخله به تفکیک قبل و بعد از مداخله فیزیوتراپی چقدر است؟
9. میانگین میزان خستگی احساس شده بعد از مسافت طی شده در تست 3 دقیقه راه رفتن در گروه های مداخله و کنترل به تفکیک قبل و بعد از مداخله فیزیوتراپی در بیماران مبتلا به کوروناویروس جدید 2019 چقدر است؟
10. میانگین میزان خستگی احساس شده بعد از مسافت طی شده در تست 3 دقیقه راه رفتن در بیماران مبتلا به کوروناویروس با و بدون ترشحات اضافی ریوی حاضر در گروه مداخله به تفکیک قبل و بعد از مداخله فیزیوتراپی چقدر است؟
11. میانگین میزان تنگی نفس احساس شده توسط بیمار در گروه های مداخله و کنترل به تفکیک قبل و بعد از مداخله فیزیوتراپی در بیماران مبتلا به کوروناویروس جدید 2019 چقدر است؟
12. میانگین میزان تنگی نفس احساس شده توسط بیمار در بیماران مبتلا به کوروناویروس با و بدون ترشحات اضافی ریوی حاضر در گروه مداخله به تفکیک قبل و بعد از مداخله فیزیوتراپی چقدر است؟
13. میانگین شاخص کیفیت زندگی SF-36 (بصورت کلی و هرکدام از دامین ها) در بازه های زمانی یک ماه پس از اتمام مداخلات در گروه های مداخله و کنترل در بیماران مبتلا به کوروناویروس جدید 2019 چقدر است؟
14. میانگین شاخص کیفیت زندگی SF-36 (بصورت کلی و هرکدام از دامین ها) در بازه های زمانی یک ماه پس از اتمام مداخلات در بیماران مبتلا به کوروناویروس با و بدون ترشحات اضافی ریوی حاضر در گروه مداخله به تفکیک قبل و بعد از مداخله فیزیوتراپی چقدر است؟
15. درصد ایجاد مرگ و میر مرتبط در گروه های مداخله و کنترل تا فاصله یک ماه پس از اعمال مداخله در بیماران مبتلا به کوروناویروس جدید 2019 چقدر است؟
16. درصد ایجاد مرگ و میر مرتبط در گروه های مداخله و کنترل تا فاصله یک ماه پس از اعمال مداخله در بیماران مبتلا به کوروناویروس با و بدون ترشحات اضافی ریوی حاضر در گروه مداخله به تفکیک قبل و بعد از مداخله فیزیوتراپی چقدر است؟

**7-5. اهداف اختصاصی (توصیفی و تحلیلی)**

**اهداف توصیفی:**

1. تعیین میانگین فشار گازهای اکسیژن و دی کسید کربن، بی کربنات، PH و درصد اشباع اکسیژن خون وریدی قبل و بعد از اعمال مداخلات به تفکیک در گروه های مداخله (فیزیوتراپی تنفسی و اسیپیرومتری تشویقی) و کنترل (اسپیرومتری تشویقی به تنهایی) در بیماران بستری مبتلا به کووید-19
2. تعیین میانگین فشار گازهای اکسیژن و دی کسید کربن، بی کربنات، PH و درصد اشباع اکسیژن خون وریدی قبل و بعد از اعمال مداخلات به تفکیک در بیماران بستری مبتلا به کووید-19 با و بدون ترشحات ریوی حاضر در گروه مداخله
3. تعیین میانگین درصد اشباع اکسیژن خونی پس از دو دقیقه استنشاق هوای آزاد قبل و بعد از اعمال مداخلات به تفکیک در گروه های مداخله (فیزیوتراپی تنفسی و اسیپیرومتری تشویقی) و کنترل (اسپیرومتری تشویقی به تنهایی) در بیماران بستری مبتلا به کووید-19
4. تعیین میانگین درصد اشباع اکسیژن خونی پس از دو دقیقه استنشاق هوای آزاد قبل و بعد از اعمال مداخلات به تفکیک در بیماران بستری مبتلا به کووید-19 با و بدون ترشحات ریوی حاضر در گروه مداخله
5. تعیین میانگین درصد اشباع اکسیژن خونی پس از دو دقیقه استنشاق اکسیژن توسط Partial Rebreather قبل و بعد از اعمال مداخلات به تفکیک در گروه های مداخله (فیزیوتراپی تنفسی و اسیپیرومتری تشویقی) و کنترل (اسپیرومتری تشویقی به تنهایی) در بیماران بستری مبتلا به کووید-19
6. تعیین میانگین درصد اشباع اکسیژن خونی پس از دو دقیقه استنشاق اکسیژن توسط Partial Rebreather قبل و بعد از اعمال مداخلات به تفکیک در بیماران بستری مبتلا به کووید-19 با و بدون ترشحات ریوی حاضر در گروه مداخله
7. تعیین میانگین مسافت طی شده در تست 3 دقیقه راه رفتن قبل و بعد از اعمال مداخلات به تفکیک در گروه های مداخله (فیزیوتراپی تنفسی و اسیپیرومتری تشویقی) و کنترل (اسپیرومتری تشویقی به تنهایی) در بیماران بستری مبتلا به کووید-19
8. تعیین میانگین مسافت طی شده در تست 3 دقیقه راه رفتن قبل و بعد از اعمال مداخلات به تفکیک در بیماران بستری مبتلا به کووید-19 با و بدون ترشحات ریوی حاضر در گروه مداخله
9. تعیین میانگین میزان خستگی احساس شده بعد از مسافت طی شده در تست 3 دقیقه راه رفتن قبل و بعد از اعمال مداخلات به تفکیک در گروه های مداخله (فیزیوتراپی تنفسی و اسیپیرومتری تشویقی) و کنترل (اسپیرومتری تشویقی به تنهایی) در بیماران بستری مبتلا به کووید-19
10. تعیین میانگین میزان خستگی احساس شده بعد از مسافت طی شده در تست 3 دقیقه راه رفتن قبل و بعد از اعمال مداخلات به تفکیک در بیماران بستری مبتلا به کووید-19 با و بدون ترشحات ریوی حاضر در گروه مداخله
11. تعیین میانگین میزان تنگی نفس قبل و بعد از اعمال مداخلات به تفکیک در گروه های مداخله (فیزیوتراپی تنفسی و اسیپیرومتری تشویقی) و کنترل (اسپیرومتری تشویقی به تنهایی) در بیماران بستری مبتلا به کووید-19
12. تعیین میانگین میزان تنگی نفس قبل و بعد از اعمال مداخلات به تفکیک در بیماران بستری مبتلا به کووید-19 با و بدون ترشحات ریوی حاضر در گروه مداخله
13. تعیین میانگین شاخص کیفیت زندگی SF-36 (بصورت کلی و هرکدام از دامین ها) یک ماه بعد از اعمال مداخلات در گروه های مداخله (فیزیوتراپی تنفسی و اسیپیرومتری تشویقی) و کنترل (اسپیرومتری تشویقی به تنهایی) در بیماران بستری مبتلا به کووید-19
14. تعیین میانگین شاخص کیفیت زندگی SF-36 (بصورت کلی و هرکدام از دامین ها) یک ماه بعد از اعمال مداخلات در بیماران بستری مبتلا به کووید-19 با و بدون ترشحات ریوی حاضر در گروه مداخله
15. درصد بروز مرگ و میر تا یک ماه بعد از اعمال مداخلات در گروه های مداخله (فیزیوتراپی تنفسی و اسیپیرومتری تشویقی) و کنترل (اسپیرومتری تشویقی به تنهایی) در بیماران بستری مبتلا به کووید-19
16. درصد بروز مرگ و میر تا یک ماه بعد از اعمال مداخلات در بیماران بستری مبتلا به کووید-19 با و بدون ترشحات ریوی حاضر در گروه مداخله

**اهداف تحلیلی:**

1. مقایسه میانگین میزان فشار گازهای اکسیژن و دی کسید کربن،PH، و درصد اشباع اکسیژن خون وریدی قبل و بعد از اعمال مداخلات در هر یک از گروه های مداخله (فیزیوتراپی تنفسی) و کنترل در بیماران بستری مبتلا به کووید-19 با قرار دادن اندازه همان متغیر در نوبت ارزیابی اول بعنوان کوواریانس
2. مقایسه میانگین میزان فشار گازهای اکسیژن و دی کسید کربن،PH، و درصد اشباع اکسیژن خون وریدی قبل و بعد از اعمال مداخلات بین بیماران با و بدون ترشحات اضافی ریوی و بستری مبتلا به کووید-19 حاضر در گروه مداخله
3. مقایسه میانگین درصد اشباع اکسیژن اکسیژن خونی پس از دو دقیقه استنشاق هوای آزاد قبل و بعد از اعمال مداخلات در هر یک از گروه های مداخله (فیزیوتراپی تنفسی) و کنترل در بیماران بستری مبتلا به کووید-19 با قرار دادن اندازه همان متغیر در نوبت ارزیابی اول بعنوان کوواریانس
4. مقایسه میانگین درصد اشباع اکسیژن اکسیژن خونی پس از دو دقیقه استنشاق هوای آزاد قبل و بعد از اعمال مداخلات بین بیماران با و بدون ترشحات اضافی ریوی و بستری مبتلا به کووید-19 حاضر در گروه مداخله
5. مقایسه میانگین درصد اشباع اکسیژن اکسیژن خونی پس از دو دقیقه استنشاق Partial Rebreather قبل و بعد از اعمال مداخلات در هر یک از گروه های مداخله (فیزیوتراپی تنفسی) و کنترل در بیماران بستری مبتلا به کووید-19 با قرار دادن اندازه همان متغیر در نوبت ارزیابی اول بعنوان کوواریانس
6. مقایسه میانگین درصد اشباع اکسیژن اکسیژن خونی پس از دو دقیقه استنشاق Partial Rebreather قبل و بعد از اعمال مداخلات بین بیماران با و بدون ترشحات اضافی ریوی و بستری مبتلا به کووید-19 حاضر در گروه مداخله
7. مقایسه میانگین میزان مسافت طی شده در تست 3 دقیقه راه رفتن قبل و بعد از اعمال مداخلات در هر یک از گروه های مداخله (فیزیوتراپی تنفسی) و کنترل در بیماران بستری مبتلا به کووید-19 با قرار دادن اندازه همان متغیر در نوبت ارزیابی اول بعنوان کوواریانس
8. مقایسه میانگین میزان مسافت طی شده در تست 3 دقیقه راه رفتن قبل و بعد از اعمال مداخلات بین بیماران با و بدون ترشحات اضافی ریوی و بستری مبتلا به کووید-19 حاضر در گروه مداخله
9. مقایسه میانگین میزان خستگی احساس شده بعد از مسافت طی شده در تست 3 دقیقه راه رفتن قبل و بعد از اعمال مداخلات در هر یک از گروه های مداخله (فیزیوتراپی تنفسی) و کنترل در بیماران بستری مبتلا به کووید-19 با قرار دادن اندازه همان متغیر در نوبت ارزیابی اول بعنوان کوواریانس
10. مقایسه میانگین میزان خستگی احساس شده بعد از مسافت طی شده در تست 3 دقیقه راه رفتن قبل و بعد از اعمال مداخلات بین بیماران با و بدون ترشحات اضافی ریوی و بستری مبتلا به کووید-19 حاضر در گروه مداخله
11. مقایسه میانگین میزان تنگی نفس احساس شده توسط بیمار قبل و بعد از اعمال مداخلات در هر یک از گروه های مداخله (فیزیوتراپی تنفسی) و کنترل در بیماران بستری مبتلا به کووید-19 با قرار دادن اندازه همان متغیر در نوبت ارزیابی اول بعنوان کوواریانس
12. مقایسه میانگین میزان تنگی نفس احساس شده توسط بیمار قبل و بعد از اعمال مداخلات بین بیماران با و بدون ترشحات اضافی ریوی و بستری مبتلا به کووید-19 حاضر در گروه مداخله
13. مقایسه شاخص کیفیت زندگی SF-36 (بصورت کلی و هرکدام از دامین ها) پس از اعمال مداخلات در هر یک از گروه های مداخله (فیزیوتراپی تنفسی) و کنترل در بیماران بستری مبتلا به کووید-19 با قرار دادن اندازه همان متغیر در نوبت ارزیابی اول بعنوان کوواریانس
14. مقایسه شاخص کیفیت زندگی SF-36 (بصورت کلی و هرکدام از دامین ها) پس از اعمال مداخلات بین بیماران با و بدون ترشحات اضافی ریوی و بستری مبتلا به کووید-19 حاضر در گروه مداخله
15. مقایسه نسبت بروز مرگ و میر پس از اعمال مداخلات در هر یک از گروه های مداخله (فیزیوتراپی تنفسی) و کنترل در بیماران بستری مبتلا به کووید-19 با قرار دادن اندازه همان متغیر در نوبت ارزیابی اول بعنوان کوواریانس
16. مقایسه نسبت بروز مرگ و میر پس از اعمال مداخلات بین بیماران با و بدون ترشحات اضافی ریوی و بستری مبتلا به کووید-19 حاضر در گروه مداخله

**9-5. نوع مطالعه**

کارآزمایی بالینی کنترل شده یک سویه کور و تدوین پروتکل درمانی

**10-5. جمعیت مورد مطالعه**

جمعیت این مطالعه بیماران بستری در بیمارستان امام خمینی بدلیل پنومونی ناشی از ابتلا به کوروناویروس جدید 2019 خواهند بود.

**11-5. روش نمونه گیری و تعیین حجم نمونه**

روش نمونه گیری در این مطالعه از طریق روش آسان و در دسترس خواهد بود.

حجم نمونه این مطالعه بر اساس مطالعه پایلوتی که توسط این گروه بر روی 10 بیمار (پنج نفر در هر گروه) اجرا شده، محاسبه گردیده است. بدین منظور اصلی ترین معیار اندازه گیری (درصد اشباع اکسیژن خونی در شرایط بدون مصرف اکسیژن) بعنوان Primary Outcome Measurement لحاظ گردیده است.

برای محاسبه حجم نمونه از فرمول زیر استفاده شد.

$$n=\frac{\left[ z_{1}-\frac{\alpha}{2}+z_{1}-\beta\right]^{2}\left( \sigma_{1}^{2}+\sigma_{2}^{2} \right)}{\left( \mu_{1}-\mu_{2} \right)^{2}}$$

α: 0.05

β: 0.1

σ_1_: 8.8

σ_2_: 8.8

μ_1-μ_2: 4%

n ≈ 13

با توجه به احتمال بالای خروج از مطالعه بنظر می رسد که نیاز است 40٪ به حجم نمونه فوق اضافه شود. بنابراین عدد **20** بعنوان حجم نمونه برای هر گروه درنظر گرفته خواهد شد.

**12-5. روش اجرای طرح**

پس از اخذ مجوز کمیته اخلاق دانشگاه علوم پزشکی تهران، با مراجعه به بخش های بستری پیش مراقبت ویژه بیماران مبتلا به کووید-19 در بیمارستان امام خمینی (ره) از بیماران با تشخیص قطعی کوروناویروس جدید 2019 و علائم بالینی Severe که غیر اینتیوبه باشند، جهت مشارکت در این طرح تحقیقاتی دعوت بعمل می آید.پس از کسب رضایت آگاهانه از بیمار، روند ارزیابی بیمار شروع می شود. در این روند تمام متغیرهای اولیه شامل آنالیز گازهای خون وریدی، درصد اشباع اکسیژن خونی در شراط استنشاق هوای آزاد و استنشاق بوسیله Partial Rebreather، میزان مسافت طی شده در تست 3 دقیقه راه رفتن و سختی پس از آن و میزان تنگی نفس از بیمار اندازه گیری خواهد شد. پیش از اعمال مداخله، پژوهشگر روند تصادفی سازی را از طریق پاکت های دربسته ای که در اختیار او قرار داده می شود، انجام می دهد. در مطالعه حاضر، روند تصادفی سازی از طریق روش Block balanced ran صورت خواهد گرفت. این روش تصادفی سازی، بهترین روش برای همگن سازی گروه های مطالعه است. در این روش ما اطمینان داریم که در هر مرحله از نمونه گیری تعداد افرادی که وارد گروه های مختلف شده اند، تا حد زیادی به هم نزدیک هستند. در ابتدا یک محقق (غیر از تراپیست) تمام حالت های ممکن چینش aaabbb را بر در نظر گرفته (مجموعا 48 حالت) و با استفاده از اعداد تصادفی 9 بلوک را بصورت تصادفی انتخاب کرده و اجزای آن ها را پشت سر هم می نویسد. سپس هر کدام از این اجزا درون یک پاکت دربسته قرار خواهد گرفت و شماره 1 الی 54 بر روی پاکت ثبت خواهد شد. تراپیست بصورت نوبتی پاکت ها را برداشته و بر مبنای آن قرار گرفتن بیمار را در گروه مداخله یا کنترل تعیین می کند (34). از آنجایی که تا زمان اجرای مداخله تراپیست از نحوه اختصاص گروه ها مطلع نیست، بنابراین ویژگی مهم Allocation concealment مطالعات RCT در این مطالعه رعایت می شود (35).

**معیارهای ورود و خروج:**

**معیارهای ورود:**

- بیماران مبتلا به کوروناویروس جدید 2019 با تشخیص قطعی پنومونی بر مبنای یافته های تصویربرداری و تست RT-PCR که در بخش های بیمارستانی بستری باشند.
- سن بین 18 تا 75 سال
- بیمار Intubated نباشد.
- بیمار هوشیار باشد و نسبت به زمان و مکان خود آگاه باشد.
- توانایی راه رفتن
- توانایی خواندن و نوشتن
- O_2_ Saturation< 88% در زمان استراحت بدون استفاده از اکسیژن

**معیارهای خروج از مطالعه:**

- وجود اختلالات عضلانی-اسکلتی بگونه­ای که مانع توانایی بیمار در برنامه فیزیوتراپی شود.
- اینتیوبه شدن بیمار در زمان اجرای مداخلات
- عدم رضایت بیمار به ادامه مشارکت در مطالعه به هر دلیلی
- عدم مشارکت در حداقل سه جلسه فیزیوتراپی

مدت زمان ارائه مداخلات در این مطالعه سه روز خواهد بود. بیماران قبل و بعد از مداخلات ارزیابی خواهند شد. همچنین یک ماه پس از اتمام دوره ارائه مداخله، با بیماران جهت ارزیابی طولانی مدت و پیگیری آن ها تماس گرفته خواهد شد.

**مداخلات گروه کنترل:**

بیماران گروه کنترل صرفا یکبار تحت مشاوره فیزیوتراپی قرار خواهند گرفت. در این مشاوره، فیزیوتراپیست به بیمار در ارتباط با نحوه سرفه موثر و تنفس عمیق توضیح خواهد داد. همچنین به این بیماران اسپیرومتر تشویقی و نحوه استفاده از آن ارائه خواهد شد.

**مداخلات گروه Experimental:**

در این فاز بیماران بصورت دوبار در روز (مجموعا شش جلسه) تحت فیزیوتراپی تنفسی قرار می گیرند. روند این برنامه شامل ارزیابی بیمار، تصمیم گیری بالینی و اجرای مداخلات درمانی است (27).

1. **ارزیابی:**

- علایم حیاتی (شامل HR, RR, BP)
- ECG
- چارت دارویی
- دمای بدن
- ABG^[[8]](#footnote-8)^ یا VBG^[[9]](#footnote-9)^
- صداهای ریوی
- لمس ریوی
- میزان درصد اشباع اکسیژن خونی
- تصویربرداری های موجود (X-Ray ویا CT-Scan)

1. **تصمیم گیری بالینی:**

با توجه به یافته ها و معاینه بالینی تراپیست در هر جلسه، دو لاین درمانی برای بیمار در نظر گرفته خواهد شد:

- **بیماران دارای ترشحات مجاری هوایی:**

در این دسته از بیماران، در ابتدا تکنیک های تخلیه ترشحات تنفسی بر مبنای تشخیص تراپیست و تصمیم گیری بالینی او اجرا خواهد شد. این درمان تا جایی ادامه دار خواهد بود که علائم وجود ترشحات مجاری تنفسی، بهبودی قابل توجهی را نشان دهد. پس از اجرای این تکنیک ها، بیمار تحت درمان های Breathing Hold و تنفس دیافراگماتیک را با دوز اشاره شده در بیماران بدون ترشحات تنفسی، خواهد رفت.

این تکنیک ها شامل Postural Drainage, Vibration, Percution, Active Cycle of Breathing (ACBT) و Cough Technique ها خواهد بود (27).

- **بیماران بدون ترشحات مجاری تنفسی:**

در این دسته از بیماران جهت کاهش میزان سفتی ایجاد شده در بافت آلوئول (بر طبق مکانیسم احتمالی مطرح شده)، تکنیک های حبس تنفس با تمرکز بر تنفس دیافراگماتیک در قالب 3 ست با 10 تکرار اجرا خواهد شد:

- ست اول: 3 ثانیه نگه داشتن، 6 ثانیه استراحت
- ست دوم: 6 ثانیه نگه داشتن، 12 ثانیه استراحت
- ست سوم: 10 ثانیه نگه داشتن، 20 ثانیه استراحت

از جلسه سوم به بعد از از بیمار خواسته می شود در انتهای درمان های دریافت شده خود، حداکثر به مدت 6 دقیقه در محیط بخش (با یا بدون اکسیژن) پیاده روی کند. فاکتورهایی که انتهای بازه پیاده روی را مشخص می کنند، به شرح ذیل می باشند:

1. O_2_ Sat به 80 درصد برسد.
2. Rated Perceived Exertion بر مبنای Borg Scale در حدفاصل 13-11 (36)

در انتها نیز به بیمار آموزش هایی در ارتباط با سرفه موثر (در صورت وجود ترشحات)، ریلکسیشن عضلات تنفسی فوقانی و تنفس دیافراگماتیک ارائه خواهد شد.

**13-5. روش و ابزار گردآوری داده ها**

**متغیرهای اولیه:**

- آنالیز گاز وریدی (VBG): برای این منظور از ورید محیطی اندام فوقانی خون گیری خواهد شد. نمونه گیری خونی بعد از 2 دقیقه بعد از استنشاق هوای آزاد انجام خواهد گرفت. فاکتورهای Pvo2, Pvco2, O2 Saturation, PH از این آنالیز استخراج خواهد شد. دلیل عدم استفاده از آنالیز گاز شریانی در این مطالعه این است که بیماران هدف ما در بخش های مراقبت ویژه بستری نیستند و بنابراین لاین شریانی ندارند، اخذ آنالیز گاز شریانی منوط به اجرای یک روند دردناک است که از نظر اخلاقی توجیهی ندارد. علاوه بر آن بیماران ما دارای یک اختلال تنفسی، و نه قلبی، هستند که بر طبق مطالعات گذشته نشان داده است بین پارامتر های آنایز گاز وریدی و شریانی، همبستگی بالای معنی داری وجود دارد (30).
- تست 3 دقیقه راه رفتن: این تست بر مبنای مطالعات انجام شده یک روش ساده و کم هزینه با شدت submaximal است که در فاز بستری و سرپایی قابل انجام بوده و همبستگی معناداری بین نتایج این تست با تست ۶ دقیقه راه رفتن مشاهده شده است (۱۹-۲۰).

برای این منظور از بیمار خواسته می شود در مدت زمان ۳ دقیقه از ابتدا تا انتهای این مسیر صاف را با حداکثر سرعتی که می تواند، راه برود. برای اجرای این تست بیمار باید کفش یا صندل راحت داشته باشد. همچنین به بیمار گفته می شود در صورتی که با هرگونه درد، ناتوانی، تنگی نفس یا سایر علایم مواجه شد، روند راه رفتن را متوقف کند و ارزیاب را آگاه کند. همچنین حین راه رفتن هیچ گونه تشویقی برای رسیدن به سرعت بیشتر از سوی ارزیاب به بیمار ارائه نمی شود. پس از اتمام تست از بیمار خواسته می شود بایستد و سپس محقق با استفاده از یک متر لیزی با دقت 0.01 متر مسیر طی شده را اندازه گیری می کند. همچنین میزان RPE بیمار بر مبنای Borg Scale بعد از این تست از وی سوال خواهد شد. در طی این مسیر تراپیست بصورت آنلاین و با استفاده از پالس انگشتی، میزان درصد اشباع اکسیژن خونی را اندازه گیری کرده و در صورتی که به کمتر از 80% برسد، بیمار بلافاصله به تخت خود انتقال داده شده و اکسیژن دریافت خواهد کرد.

در صورت بروز عوارض جانبی سیستم قلبی-عروقی و یا اختلال هوشیاری حین این تست اقدامات اورژانسی توسط فیزیوتراپیست اجرا شده و از آنجایی که این اقدام در محیط بیمارستان انجام می شود، سریعا به پزشکان مقیم در بخش اطلاع داده می شود.

- میزان O2 Saturation بعد از 2 دقیقه استفاده از Partial Rebreather: در این تست از بیمار خواسته می شود به مدت دو دقیقه در پوزیشن Fowler^[[10]](#footnote-10)^ قرار بگیرد. در این زمان یک ماسک Partial Rebreather بر روی صورت او بصورت کاملا فیکس شده قرار گرفته و از وی خواسته می شود نفس های عمیق بکشد. بعد از این دو دقیقه تنفس میزان درصد اشباع اکسیژن خونی با استفاده از پالس اکسیمتر اندازه و ثبت می شود.
- میزان O2 Saturation بعد از 2 دقیقه استنشاق هوای آزاد: در این تست از یمار خواسته می شود به مدت دو دقیقه در پوزیشن Fowler قرار بگیرد و بدون مصرف اکسیژن در هوای آزاد نفس بکشد. بعد از این دو دقیقه تنفس میزان درصد اشباع اکسیژن خونی با استفاده از پالس اکسیمتر اندازه و ثبت می شود.
- میزان احساس تنگی نفس: در ابتدا یک تصویر بر مبنای Visual Analogue Scale (VAS) در اختیار بیمار قرار گرفته و به بیمار آموزش داده می شود که عدد صفر به منزله نداشتن هیچگونه تنگی نفس و عدد 10 بمنزله داشتن حداکثر تنگی نفس می باشد. از او خواسته می شود با استفاده از خودکار میزان تنگی نفس خود را در 24 ساعت گذشته بر روی کاغذ علامت گذاری کند.

**متغیرهای ثانویه:**

- Borg scale: در این مقیاس عدد ۶ به منزله داشتن تنگی نفس خیلی خفیف و عدد ۱۹ بمنزله داشتن تنگی نفس بسیار شدید در حین انجام فعالیت یا تمرینات می باشد. پس از آموزش به بیمار از وی خواسته می شود که میزان تنگی نفس خود را در حین فعالیت از روی مقیاس مشخص نماید.
- پیگیری طولانی مدت بیمار: بعد از یک ماه پس از اتمام مداخلات، با بیمار تماس گرفته خواهد شد و علاوه بر تکمیل پرسشنامه کیفیت زندگی Short form-36 از وی در ارتباط با مرگ و میر (همراهان) و بستری مجدد سوال خواهد شد. همچنین پرونده درمانی بیمار در این مدت زمان بررسی شده و علاوه بر اطلاعات فوق در ارتباط با طول مدت زمان بستری و سیر بیماری اطلاعات جمع آوری می شود.

**14-5. تدوین پروتکل درمانی**

یکی از اهداف اصلی این مطالعه تدوین یک پروتکل درمانی از جانب دانشگاه علوم پزشکی تهران در ارتباط با نحوه مدیریت (ارزیابی و درمان) بیماران بستری به دلیل پنومونی ناشی از کووید-19 است. جهت نیل به این پروتکل، پس از اتمام روند کارآزمایی بالینی یک مرور نظام مند بر روی تمام مطالعات منتشر شده در ارتباط با برنامه فیزیوتراپی این بیماران در فاز بستری ارائه خواهد شد. در این روند روش جستجو و استخراج داده کاملا نظام مند و بر طبق استاندارد PRISMA^[[11]](#footnote-11)^ خواهد بود (37). پس از استخراج داده و ارزیابی کیفیت آن مطالعات و همچنین گزارش اجرایی این کارآزمایی، نتایج آن در یک پنل خبرگان با حضور اعضای متخصص فیزیوتراپی و سایر حوزه های مرتبط پزشکی مطرح خواهد شد تا اعضای آن نظرات خود را بیان کنند. پژوهشگر اصلی مطالعه تمام نظرات را جمع آوری و دسته بنده کرده و متناسب با سطوح شواهد موجود و کیفیت آن ها ، پیش نویسی برای متن پروتکل درنظر می گیرد. این متن مجدد در جلسه خبرگان مطرح شده و از اعضای آن در ارتباط با میزان شفافیت و لزوم طرح تک تک اجزای پروتکل سوال می شود (Content Vlidity). پس از تغییرات صورت گرفته در متن پیش نویس توسط اعضای پنل، نسخه دیگری از آن تهیه شده و در اختیار پنج فیزیوتراپیست درگیر با بیماری کووید-19 قرار داده می شود. از این افراد خواسته می شود تا نظر خود را درباره میزان درک مطالب بیان کنند (Face Validity). همچنین از آن ها خواسته می شود که نظر خود را درباره بندهای غیرمفهوم بیان کنند (Face Validity). خروجی این مراحل پروتکل فیزیوتراپی در بستری بیماران مبتلا به پنومونی ناشی از کووید-19 را فراهم می آورد.

**15-5. ملاحظات اخلاقی**

- از کلیه بیماران موافقت آگاهانه گرفته خواهد شد.
- روند تحقیق، نقش بیمار و احتمال قرارگیری او در گروه مداخله یا کنترل در آن به طور شفاف توضیح داده خواهد شد.
- روش اجرای تحقیق هیچ گونه آسیب و هزینه ای برای بیمار به همراه نخواهد داشت.
- بیمار در هر مرحله ای از تحقیق آزاد بوده که به هر علتی یا بدون علت مطالعه را ترک کند و این کار لطمه ای به درمان وی وارد نمیکند.
- حفاطت از اطلاعات شخصی بیماران و اصل رازداری به طور کامل رعایت خواهد شد.
- با توجه به اینکه ممکن است بیمار از هوشیاری کافی برای درک موضوع جهت مشارکت در طرح نداشته باشد، صرفا بیمارانی وارد طرح خواهند شد که دارای هوشیاری کامل باشند.

**16-5. جدول زمانبندي اجرای طرح از تصويب تا دفاع نهايي**

|  | | زمان اجرا به ماه | | | | | | | | | | | | | | | | | |
| --- | --- | --- | --- | --- | --- | --- | --- | --- | --- | --- | --- | --- | --- | --- | --- | --- | --- | --- | --- |
| رديف | فعاليتهاي اجرائي | زمان كل |  |  |  |  |  |  |  |  |  |  |  |  |  |  |  |  |  |
| 1 | جمع آوری اطلاعات | 1ماه | * |  |  |  |  |  |  |  |  |  |  |  |  |  |  |  |  |
| 2 | طراحی پروژه مقدماتی و اجرای آن | 1 ماه | * |  |  |  |  |  |  |  |  |  |  |  |  |  |  |  |  |
| 3 | نمونه گیری و اجرای مداخله | 5 ماه |  | * | * | * | * | * |  |  |  |  |  |  |  |  |  |  |  |
| 4 | بررسی آماری و تجزیه و تحلیل | 1 ماه |  |  | * |  |  |  | * |  |  |  |  |  |  |  |  |  |  |
| 5 | نگارش مقاله و کنترل نهایی | 2 ماه |  |  |  |  |  |  | * | * |  |  |  |  |  |  |  |  |  |

**17-5. پیوست ها**

**پرسشنامه SF-36:**

| سوالات زیر به منظور بررسی نظر شما در مورد وضعیت سلامتی‌تان طراحی شده است. لطفا هر سوال را با ضربدر زدن(×)پاسخ دهید. | | | |
| --- | --- | --- | --- |
| 1. به طور کلی وضعیت سلامت خود را چگونه می بینید؟   عالی خیلی خوب خوب متوسط ضعیف | | | |
| 1. میزان سلامتی خود را در حال حاضر با مقایسه با سال قبل چگونه ارزیابی می کنید؟   الف)از سال قبل بسیار بهتر هستم ب)تا حدی بهتر از سال قبل هستم ج)مثل سال قبل هستم  د) در حال حاضر تا حدی بدتر از سال قبل هستم ه) در حال حاضر بسیار بدتر از سال قبل هستم | | | |
| - سوالاتی که در زیر می‌آید درباره‌ی فعا لیتهایی است که شما به طور عادی در طول روز انجام می دهید آیا در انجام این   این فعالیتها محدودیت یا مشکلی دارید؟ اگر دارید چقدر است؟ (لطفا یک مورد را علامت بزنید): | | | |
| سوال | مشکل دارم | کمی مشکل دارم | اصلا مشکل ندارم |
| 3-در فعالیتهای شدید مثل دویدن، بلند کردن اجسام سنگین، شرکت کردن در ورزش های سنگین |  |  |  |
| 4- درفعالیتهای متوسط مثل جا به جا کردن میز، کشیدن جارو برقی و... |  |  |  |
| 5- در حمل کردن خرید روزانه |  |  |  |
| 6- در بالا رفتن از چند طبقه |  |  |  |
| 7- در بالا رفتن از یک طبقه |  |  |  |
| 8- خم شدن، دولا شدن، زانو زدن |  |  |  |
| 9- پیاده روی بیش از یک کیلومتر |  |  |  |
| 10- پیاده روی به فاصله چند صد متر |  |  |  |
| 11- پیاده روی به فاصله یک صد متر |  |  |  |
| 12- در استحمام یا پوشیدن لباسهای خود |  |  |  |

| - در طی چهارهفته گذشته آیا در کار یا تنظیم فعا لیت روزانه خود مشکلات زیر را به خاطر مشکل جسمی داشته اید؟   بلی خیر | | |
| --- | --- | --- |
| 13- مجبور شده اید که از زمان لازم برای انجام کار یا فعالیت های دیگرکم کنید؟ |  |  |
| 14- کمتر از آنچه که میخواستید وقت گذاشته اید؟ |  |  |
| 15- در انجام یک نوع کار یا فعالیت خاص مشکل داشته اید؟ |  |  |
| 16- کار عادی خود را با سختی و مشکل بیشتری انجام داده اید؟ |  |  |

| - در طی چهار هفته گذشته به علت مسائل روانی مثل اضطراب و یا افسردگی در انجام امور معمولی روزانه و انجام کارها   با مشکلات زیر روبه رو بوده اید؟  بلی خیر | | |
| --- | --- | --- |
| 17- مجبور شده اید که از زمان لازم برای انجام کار یا فعالیت های دیگرکم کنید؟ |  |  |
| 18- کمتر از آنچه که میخواستید وقت گذاشته اید؟ |  |  |
| 19- در انجام کارهای روزمره از دقت معمول برخوردار نبوده اید؟ |  |  |

| 20- درطی چهار هفته گذشته وضعیت سلامت جسمی و روانی شما تا چه اندازه در روابط اجتماعی معمول شما در رابطه با  خانواده ، دوستان، همسایگان و یا سایر افراد اختلال ایجاد کرده است؟  هیچ به طور جزئی نسبتا متوسط مقداری فوق العاده زیاد |
| --- |
| 21- در طی چهار هفته گذشته چه مقداردرد بدنی داشته اید؟  هیچ خیلی خفیف خفیف متوسط شدید خیلی شدید |
| 22- در طی چهار هفته گذشته درد بدنی تا چه اندازه مانع از انجام کارهای عادی شما شده است؟  اصلا مانع نشده کمی به طور متوسط تقریبا تا حدی فوق العاده زیاد |

| - این سوالها در مورد احساس شما و اینکه در طی چهار هفته گذشته چطور بودید، است لطفا موردی را انتخاب کنید که به   احساس شما نزیکتر باشد. | | | | | | |
| --- | --- | --- | --- | --- | --- | --- |
| گزینه‌ها: | همه اوقات | بیشتر اوقات | مقدار زیادی از اوقات | گاهی اوقات | مقدارکمی از ا وقات | هیچ وقت |
| 23- آیا روحیه خوبی داشته اید؟ |  |  |  |  |  |  |
| 24- آیا خیلی عصبانی بوده اید؟ |  |  |  |  |  |  |
| 25- آیا آنقدر عصبانی بوده اید که هیچ چیز شما را خوشحال نمی کرد؟ |  |  |  |  |  |  |
| 26- آیا احساس آرامش می کردید؟ |  |  |  |  |  |  |
| 27- آیا خود را با انرژی احساس می کردید؟ |  |  |  |  |  |  |
| 28- آیا احساس فرسودگی داشته اید؟ |  |  |  |  |  |  |
| 29- آیا احساس غمگینی یا نا امیدی داشته اید؟ |  |  |  |  |  |  |
| 30- آیا شخص خوشحالی بوده اید؟ |  |  |  |  |  |  |
| 31- آیا احساس خستگی داشتید؟ |  |  |  |  |  |  |

| 32-در طی چهارهفته گذشته تا چه میزان مشکلات و مسائل جسمی و عاطفی بر فعالیتهای اجتماعی شما تاثیرگذار بوده است؟  همه ی اوقات بیشتر اوقات گاهی اوقات مقدار کمی از اوقات هیچ وقت |
| --- |

| - موارد زیر تا چه اندازه در مورد شما صادق یا نادرست است. | | | | | |
| --- | --- | --- | --- | --- | --- |
| گزینه‌ها: | کاملا درست | بیشتر درست | نمیدانم | بیشتر نادرست | کاملا نا درست |
| 33- من از دیگران زودتر بیمار می شوم |  |  |  |  |  |
| 34- سلامت من در حد سلامت دیگران است |  |  |  |  |  |
| 35- انتظار دارم وضعیت سلامتی ام بدتر شود |  |  |  |  |  |
| 36- وضعیت سلامتی من در حد عالی است |  |  |  |  |  |

**18-5. فهرست منابع**

1. Zhu N, Zhang D, Wang W, Li X, Yang B, Song J, et al. A novel coronavirus from patients with pneumonia in China, 2019. New England Journal of Medicine. 2020.

2. Read JM, Bridgen JR, Cummings DA, Ho A, Jewell CP. Novel coronavirus 2019-nCoV: early estimation of epidemiological parameters and epidemic predictions. MedRxiv. 2020.

3. Huang C, Wang Y, Li X, Ren L, Zhao J, Hu Y, et al. Clinical features of patients infected with 2019 novel coronavirus in Wuhan, China. The lancet. 2020;395(10223):497-506.

4. Zhou M, Zhang X, Qu J. Coronavirus disease 2019 (COVID-19): a clinical update. Frontiers of medicine. 2020:1-10.

5. Chen J. Pathogenicity and transmissibility of 2019-nCoV—a quick overview and comparison with other emerging viruses. Microbes and infection. 2020.

6. Liu T, Hu J, Kang M, Lin L, Zhong H, Xiao J, et al. Transmission dynamics of 2019 novel coronavirus (2019-nCoV). 2020.

7. Li Q, Guan X, Wu P, Wang X, Zhou L, Tong Y, et al. Early transmission dynamics in Wuhan, China, of novel coronavirus–infected pneumonia. New England Journal of Medicine. 2020.

8. Tang CY, Taylor NF, Blackstock FC. Chest physiotherapy for patients admitted to hospital with an acute exacerbation of chronic obstructive pulmonary disease (COPD): a systematic review. Physiotherapy. 2010 Mar 1;96(1):1-3.

9. Wujtewicz M, Dylczyk-Sommer A, Aszkiełowicz A, Zdanowski S, Piwowarczyk S, Owczuk R. COVID-19–what should anaethesiologists and intensivists know about it? Anaesthesiology intensive therapy. 2020;52(1):34-41.

10. Xu Z, Shi L, Wang Y, Zhang J, Huang L, Zhang C, et al. Pathological findings of COVID-19 associated with acute respiratory distress syndrome. The Lancet respiratory medicine. 2020;8(4):420-2.

11. Franks TJ, Chong PY, Chui P, Galvin JR, Lourens RM, Reid AH, et al. Lung pathology of severe acute respiratory syndrome (SARS): a study of 8 autopsy cases from Singapore. Human pathology. 2003;34(8):743-8.

12. Zhang H, Kang Z, Gong H, Xu D, Wang J, Li Z, et al. The digestive system is a potential route of 2019-nCov infection: a bioinformatics analysis based on single-cell transcriptomes. BioRxiv. 2020.

13. Baig AM. Computing the Effects of SARS-CoV-2 on Respiration Regulatory Mechanisms in COVID-19. ACS Chemical Neuroscience. 2020.

14. . !!! INVALID CITATION !!! (12, 13).

15. Mirastschijski U, Dembinski R, Maedler K. Lung Surfactant for Pulmonary Barrier Restoration in Patients With COVID-19 Pneumonia. Frontiers in Medicine. 2020;7:254.

16. Jin X, Lian J-S, Hu J-H, Gao J, Zheng L, Zhang Y-M, et al. Epidemiological, clinical and virological characteristics of 74 cases of coronavirus-infected disease 2019 (COVID-19) with gastrointestinal symptoms. Gut. 2020;69(6):1002-9.

17. Thomas P, Baldwin C, Bissett B, Boden I, Gosselink R, Granger CL, et al. Physiotherapy management for COVID-19 in the acute hospital setting: clinical practice recommendations. Journal of Physiotherapy. 2020.

18. Liu K, Zhang W, Yang Y, Zhang J, Li Y, Chen Y. Respiratory rehabilitation in elderly patients with COVID-19: A randomized controlled study. Complementary therapies in clinical practice. 2020:101166.

19. Ambrosino N, Foglio K, Rubini F, Clini E, Nava S, Vitacca M. Non-invasive mechanical ventilation in acute respiratory failure due to chronic obstructive pulmonary disease: correlates for success. Thorax. 1995;50(7):755-7.

20. Munshi L, Kobayashi T, DeBacker J, Doobay R, Telesnicki T, Lo V, et al. Intensive care physiotherapy during extracorporeal membrane oxygenation for acute respiratory distress syndrome. Annals of the American Thoracic Society. 2017;14(2):246-53.

21. Yang F, Liu N, Hu J, Wu L, Su G, Zhong N, et al. Pulmonary rehabilitation guidelines in the principle of 4S for patients infected with 2019 novel coronavirus (2019-nCoV). Zhonghua jie he he hu xi za zhi= Zhonghua jiehe he huxi zazhi= Chinese journal of tuberculosis and respiratory diseases. 2020;43(3):180-2.

22. Wang TJ, Chau B, Lui M, Lam G-T, Lin N, Humbert S. PM&R and Pulmonary Rehabilitation for COVID-19. American Journal of Physical Medicine & Rehabilitation. 2020.

23. Iannaccone S, Castellazzi P, Tettamanti A, Houdayer E, Brugliera L, de Blasio F, et al. Role of Rehabilitation Department for Adult Individuals With COVID-19: The Experience of the San Raffaele Hospital of Milan. Archives of Physical Medicine and Rehabilitation. 2020.

24. Lazzeri M, Lanza A, Bellini R, Bellofiore A, Cecchetto S, Colombo A, et al. Respiratory physiotherapy in patients with COVID-19 infection in acute setting: a Position Paper of the Italian Association of Respiratory Physiotherapists (ARIR). Monaldi Archives for Chest Disease. 2020;90(1).

25. Yang L-L, Yang T. Pulmonary rehabilitation for patients with coronavirus disease 2019 (COVID-19). Chronic Diseases and Translational Medicine. 2020.

26. Simpson R, Robinson L. Rehabilitation After Critical Illness in People With COVID-19 Infection. American journal of physical medicine & rehabilitation. 2020;99(6):470.

27. Hillegass E. Essentials of Cardiopulmonary Physical Therapy-E-Book: Elsevier Health Sciences; 2016.

28. of the International CSG. The species Severe acute respiratory syndrome-related coronavirus: classifying 2019-nCoV and naming it SARS-CoV-2. Nature Microbiology. 2020;5(4):536.

29. Theodore AC. Venous blood gases and other alternatives to arterial blood gases. Up to Date. 2019.

30. Ak A, Ogun CO, Bayir A, Kayis SA, Koylu R. Prediction of arterial blood gas values from venous blood gas values in patients with acute exacerbation of chronic obstructive pulmonary disease. The Tohoku journal of experimental medicine. 2006;210(4):285-90.

31. Bohannon RW, Bubela DJ, Wang Y-C, Magasi SS, Gershon RC. Six-minute walk test versus three-minute step test for measuring functional endurance (Alternative Measures of Functional Endurance). Journal of strength and conditioning research/National Strength & Conditioning Association. 2015;29(11):3240.

32. Durand F. How to improve long‐term outcome after liver transplantation? Liver International. 2018;38:134-8.

33. Hafen BB, Sharma S. Oxygen saturation. StatPearls [Internet]: StatPearls Publishing; 2019.

34. Efird J. Blocked randomization with randomly selected block sizes. International journal of environmental research and public health. 2011;8(1):15-20.

35. Doig GS, Simpson F. Randomization and allocation concealment: a practical guide for researchers. Journal of critical care. 2005;20(2):187-91.

36. Borg GA. Psychophysical bases of perceived exertion. Medicine & Science in Sports & Exercise. 1982.

37. Moher D, Liberati A, Tetzlaff J, Altman DG, Group P. Preferred reporting items for systematic reviews and meta-analyses: the PRISMA statement. PLoS med. 2009;6(7):e1000097.

1. Angiotensin-converting enzyme 2 [↑](#footnote-ref-1)
2. تفاوت CPAP و BiPAP در این است که BiPAP در تنفس بیمار دو فشار اعمال می کند؛ یک فشار در فاز دم و کمترین فشار هم در فاز بازدم ولی در CPAP ما صرفا یک فشار ثابت در تمام فاز های تنفسی بیمار خواهیم داشت. بسیاری از بیماران BiPAP را نسبت به CPAP راحت تر تحمل می کنند. [↑](#footnote-ref-2)
3. Severe Acute Respiratory Syndrome 2 [↑](#footnote-ref-3)
4. Acute Respiratory Distress Syndrome [↑](#footnote-ref-4)
5. یک ابزاری جهت تحریک سرفه بیمار جهت خروج موثر ترشحات اضافی ریوی است. [↑](#footnote-ref-5)
6. منظوراز مدیریت بین رشته ای ونتیلاسیون غیر تهاجمی همکاری نزدیک گروه های مختلف پزشکی این حوزه از جمله فیزیوتراپی جهت احیای هرچه سریعتر تنفس مستقل بیمار است. [↑](#footnote-ref-6)
7. Venous Blood Gasses (VBG) [↑](#footnote-ref-7)
8. Arterial Blood Gas [↑](#footnote-ref-8)
9. Venous Blood Gas [↑](#footnote-ref-9)
10. قرار گرفتن بیمار در وضعیت خوابیده بگونه ای که سر تخت بین 30 تا 45 درجه بالا بیاید. [↑](#footnote-ref-10)
11. Preferred Reporting Items for Systematic Reviews and Meta-Analyses [↑](#footnote-ref-11)
